# Spatiotemporal Local-Remote Senor Fusion (ST-LRSF) for Cooperative Vehicle Positioning

**DOI:** 10.3390/s18041092

**Published:** 2018-04-04

**Authors:** Han-You Jeong, Hoa-Hung Nguyen, Adhitya Bhawiyuga

**Affiliations:** 1 School of Electrical and Computer Engineering, Pusan National University, 46241 Busan, Korea; nguyenhoahungit@gmail.com; 2 Faculty of Computer Science, Brawijaya University, 65145 Malang, Indonesia; bhawiyuga@ub.ac.id

**Keywords:** vehicle sensors, cooperative vehicle positioning, sensor-data association, spatiotemporal dissimilarity

## Abstract

Vehicle positioning plays an important role in the design of protocols, algorithms, and applications in the intelligent transport systems. In this paper, we present a new framework of *spatiotemporal local-remote sensor fusion (ST-LRSF)* that cooperatively improves the accuracy of absolute vehicle positioning based on two state estimates of a vehicle in the vicinity: a local sensing estimate, measured by the on-board exteroceptive sensors, and a remote sensing estimate, received from neighbor vehicles via vehicle-to-everything communications. Given both estimates of vehicle state, the ST-LRSF scheme identifies the set of vehicles in the vicinity, determines the reference vehicle state, proposes a spatiotemporal dissimilarity metric between two reference vehicle states, and presents a greedy algorithm to compute a minimal weighted matching (MWM) between them. Given the outcome of MWM, the theoretical position uncertainty of the proposed refinement algorithm is proven to be inversely proportional to the square root of matching size. To further reduce the positioning uncertainty, we also develop an extended Kalman filter model with the refined position of ST-LRSF as one of the measurement inputs. The numerical results demonstrate that the proposed ST-LRSF framework can achieve high positioning accuracy for many different scenarios of cooperative vehicle positioning.

## 1. Introduction

An accurate estimation of real-time vehicle position is a crucial requirement for the success of many protocols, algorithms, and applications of vehicle safety and intelligent transportation systems (ITS) [1,2,3,4]. For example, in the medium-access control (MAC) layer, a vehicle at a longer distance from the sending vehicle is likely to be chosen as a relay node for message dissemination [2]. In the geographic routing algorithms, a message is forwarded to the closest neighbor vehicle to its destination [3]. In addition, limiting the uncertainty of vehicle position to the width of a lane is of utmost importance in vehicle tracking and many cooperative safety-critical applications, such as cooperative collision avoidance, lane change warning, etc. [1,4].

The Global Navigation Satellite System (GNSS), such as the Global Positioning System (GPS) or the Galileo system, is widely adopted for vehicle positioning due to the cost efficiency, the global coverage, and the availability of commodity GPS receivers in the marketplace. In this system, both the position and the reference time of a vehicle are estimated by real-time processing of radio-frequency (RF) signals sent from at least four different satellites [5]. However, it is shown that its position estimate has the uncertainty up to tens of meters, and is temporarily unavailable in harsh environments, such as tunnels, buildings, vegetations, etc. [4,5,6]. In this paper, we focus on the vehicle positioning scheme that can reduce the uncertainty of GPS position estimate.

In the last few decades, extensive studies have been devoted to accurate and reliable estimation of vehicle state, which is defined as the combination of vehicle kinematics including position, speed, and/or heading. The basic idea of this approach is the use of sequential estimator incorporating direct and indirect observations of vehicle state, which consists of the following two state estimations [7,8,9]: first, the predicted vehicle state is represented by a set of system equations in terms of the current vehicle state, the measured control inputs, and the system noises. Second, the measurement of vehicle state is also formulated by system equations with respect to the predicted state and the measurement of on-board sensors, such as the GPS, accelerometer, compass, and/or gyroscope. When a GPS outage happens, the accumulation of predicted vehicle states can be used for the position reference, which is called the dead reckoning [5,7]. When both estimates are available, the accuracy of vehicle state can be significantly improved by using a linear least-square optimal estimator, called the Kalman filter [7,8,9].

In the last decade, the automotive industry has commercialized many on-board exteroceptive sensors, such as radars, cameras, and laser scanners, for estimating the relative state of surrounding objects [10,11,12,13,14,15,16,17]. This estimate is called the local sensing estimate (LSE) in this paper. These sensors were initially introduced to the high-end cars only, currently also being mounted on the mid-range cars, and expected to be present all kinds of cars soon. The richness and high precision of these devices make it possible to estimate both relative and absolute positionings with high accuracy [10]. In detail, the relative positioning of each surrounding vehicle is a key parameter of collision risk assessment [10,11,12], whereas the absolute positioning with high-precision environment map is the essential functionality of autonomous driving [13,14,15,16]. The primary goal of this paper is to improve the accuracy of absolute positioning without high-precision environment map.

With the recent standardization of vehicle-to-everything (V2X) communications in [18,19,20,21,22], the on-board V2X communication device can be seen as a virtual sensor that cooperatively provides the proprioceptive sensing data of neighbor vehicles, such as sensing time, position, speed, heading, etc. To collect these sensing data, an on-board unit (OBU) usually has a GPS receiver and the interface to in-vehicle networks, such as the controller-area networks (CAN) [23]. Furthermore, information sharing via the V2X communications relaxes the limitations of on-board exteroceptive sensors: The V2X communication signal reaches up to one kilometer and is less susceptible to the requirement of direct visibility. In this paper, the state estimate of neighbor vehicle via the cooperative V2X communications is called the remote sensing estimate (RSE).

Given both estimates of vehicle state, it is natural to ask the following questions: *How should the framework of sensor-data fusion between these two sets of sensing estimates be designed?* and *How can the statistical characteristics of estimated pairs be exploited to improve the accuracy of vehicle positioning?* In highly dynamic vehicular environments, it is challenging to find the answers to these questions, due to the uncertainty of GPS device, no common reference of vehicle state between them, the cardinality of both estimates spanning up to a hundred of vehicles, highly dynamic connectivity and visibility conditions, and possibly incorporating indirect, inaccurate, and intermittent observations. Although a few different approaches, such as the GPS pseudorange sharing [24,25] and the crosslayer V2X positioning [26,27,28,29], have recently been introduced in the area of cooperative vehicle positioning, these research questions still remain open.

In this paper, based on our earlier works in [30,31], we present a novel framework of *spatiotemporal local-remote sensor fusion (ST-LRSF)* that improves the accuracy of absolute vehicle positioning using the statistical correction information extracted from the data association between two sets of state estimates. In order to identify the sets of surrounding and neighbor vehicles, the ST-LRSF first presents a constant-speed model to estimate the present state of missed LSE and RSE, respectively. It also determines the reference vehicle state to which both sensing estimates can be easily converted. To mitigate the instant randomness of GPS position estimate, the ST-LRSF proposes a spatiotemporal dissimilarity metric between two reference vehicle states. Then, the greedy sensor-data association (GSA) algorithm is presented to compute a minimal weighted matching (MWM) between both sets of sensing estimates. Given the outcome of MWM with cardinality *M*, the position refinement with center of mass (PRCoM) is proposed to refine the vehicle positioning whose theoretical lower bound (LB) on the uncertainty is down to $1/\sqrt{M}$ of GPS error. It is also shown that the positioning uncertainty can be further reduced by taking the refined position of ST-LRSF as a measurement input of extended Kalman filter (EKF). The numerical results show that the ST-LRSF schemes achieve the best positioning accuracy for a wide range of parameters: the standard deviation (STD) of GPS error, vehicle density, sensing range, sensing period, and vehicle speed. Our contributions are summarized as follows:

- A comprehensive model for cooperative vehicle positioning is established to clearly address the detailed characteristics of multiple different on-board sensors.
- The framework of ST-LRSF is shown to be computationally efficient, robust to the instant randomness of GPS error, adaptive to the dynamic change of estimation sets, and not incurring any additional overhead to the V2X communications.
- The spatiotemporal dissimilarity metric is proposed for the sensor-data association, and is shown to be effective for the mitigation of instant random GPS error.
- To the best of our knowledge, the PRCoM is the first approach that exploits the aggregated statistical characteristics of surrounding vehicles to improve the positioning accuracy of vehicle itself. In addition, the lower bound on the root-mean-square error (RMSE) of PRCoM is proven to be $1/\sqrt{M}$ of GPS RMSE.

The remainder of this paper is organized as follows. We first summarize the related work in Section 2, and introduce the model for cooperative vehicle positioning in Section 3. Based on this model, the cooperative vehicle positioning problem is specified in Section 4. To address this problem, we present the framework of ST-LRSF in Section 5. In Section 6, the numerical results from extensive simulations are presented and discussed. Finally, we conclude this paper in Section 7.

## 2. Related Work

The vehicle positioning approaches in the literature can be classified into two categories: the relative positioning [10,11,12,13,14] and the absolute positioning [7,8,9,15,16,24,25,26,27,28,32]. Table 1 summarizes a few different approaches to vehicle positioning. In the following sections, we discuss the distinctive characteristics of each approach, as well as the summary of related works.

### 2.1. Relative Vehicle Positioning

The relative vehicle positioning has been adopted as the position reference for the current advanced driver-assistance systems (ADAS) [10,17]. It relies on the on-board exteroceptive sensors to represent the relative state of surrounding objects, such as vehicles, motorcycles, and pedestrians, in its local coordinate. In this representation, the lack of absolute position reference is still acceptable, provided that the exteroceptive sensor can accurately track the relative states of all moving objects, called the targets. A tracking filter is usually employed to minimize the mean square error of the target state against the high mobility of vehicles, the obstruction of sensor signals, and the random reflection from road surface and environments, called the clutters [13,14,33].

The key problem of the tracking filter is how to determine the origin of each sensor measurement and how to associate it with the list of targets. For the exteroceptive sensor at a fixed position, the probabilistic data association filter (PDAF) in [13] calculates the association probability of each valid measurement to the targets being tracked by the EKF-based tracking filter whose state is expanded to include the kinematics of all targets. The key idea of the PDAF framework is to approximate all past information about the target state to the statistics in the form of Gaussian posterior, which significantly reduces the computational complexity. In [33], an unscented Kalman filter (UKF) is designed for the radar of a moving vehicle that tracks the targets by taking both radar measurements and V2X communications as the measurement inputs. An alternative approach is to use the interacting multiple models (IMM) for driving behaviors and the sequential multiple hypothesis tests for associating radar measurement and V2X communications [14]. However, the intensive computation involved in the state estimation and the data association limits its applicability to simultaneously track many surrounding vehicles. Furthermore, the relative positioning has the limitations originating from the exteroceptive sensors, when it is applied to the emerging new ITS applications: the unawareness of absolute position, the limited sensing ability due to its short sensing range usually less than 100 m, and the strict requirement of direct visibility. Therefore, our focus in this paper is on the absolute vehicle positioning.

### 2.2. Absolute Vehicle Positioning

**Figure 1:**
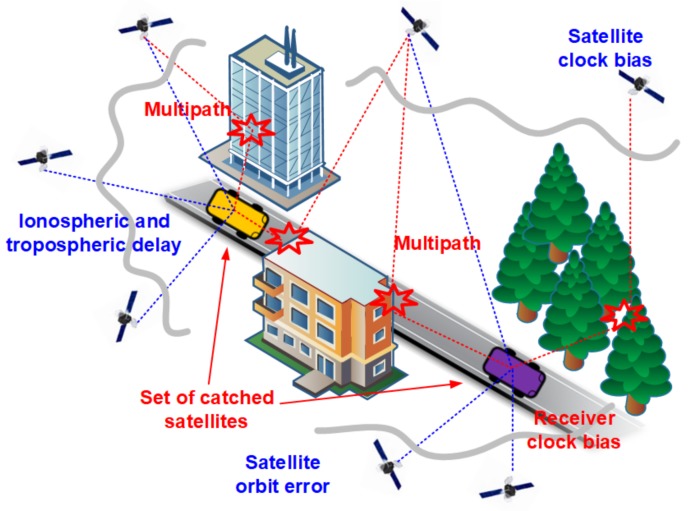
Sources of the GPS errors—The sources with blue and red texts are for bias and random errors, respectively.

Although each measurement of vehicle state suffers from the uncertainty and noise, it is obvious that the measurement error of GPS device is the dominant component of the whole information uncertainty. Figure 1 illustrates multiple sources of the GPS errors that can be classified into biases and random errors [5]. The biases are caused by a satellite orbit bias, a satellite clock bias, ionospheric and tropospheric delay, etc., and are common to all GPS receivers in a geographical area (<50 km distance) and in the correlation time around 300 s [6]. Usually, the biases are compensated by the differential correction information sent by a reference station at a known position, which is known as the differential GPS (DGPS) [5,6]. On the other hand, the sources of random errors include a multipath effect, a receiver clock error, and the difference in the set of observed satellites at each GPS receiver. Contrary to the biases, the impact of these factors on each GPS device is all different, which results in much less spatiotemporal correlation. As a result, it is very difficult to compensate the random errors using the differential correction information. In the following sections, we discuss three absolute vehicle positioning approaches: standalone, map-based, and cooperative vehicle positioning.

#### 2.2.1. Standalone Vehicle Positioning

There has been a continuing demand to enhance the accuracy and availability of absolute vehicle positioning in many ITS applications [1,2,3,4]. To achieve this goal, the papers in [7,8,9] have studied the fusion of on-board proprioceptive sensor data and GPS position fix based on the EKF model. In [7], the dead reckoning based on the proprioceptive sensors, such as odometers, compasses, gyroscopes, accelerometers, can be a good alternative to the GPS position fix when GPS is temporarily unavailable. To increase the availability of vehicle positioning in an urban canyon with high-rise buildings, an approximation of vehicle path as pieces of lines is incorporated in the EKF+IMM model in [8]. This scheme is suitable for urban canyon environments because a vehicle catching at least two satellite signals can still estimate its position. The paper in [9] presents an EKF model that fuses multiple on-board proprioceptive sensors with a DGPS position estimate based on a dynamic bicycle model. To summarize, this approach presents different variants of EKF models integrating GPS position estimate with multiple on-board proprioceptive sensing data. Consequently, the positioning accuracy strongly depends on the fidelity of underlying sensors, such as GPS device and on-board sensors.

#### 2.2.2. Map-Based Vehicle Positioning

In [15,16], the use of on-board exteroceptive sensors and a high-precision environment map makes it possible for robotic autonomous vehicle to estimate the pose, i.e., the combination of position and orientation. In this approach, the vehicle state is flexibly expanded to include the positions of extracted features from the surrounding road environments, called the landmarks. Then, a robotic vehicle updates the environment map based on the current measurement, and simultaneously finds its pose within it, which is known as the simultaneous positioning and mapping (SLAM) [15,16]. The map-based vehicle positioning in [15,16] uses a three-dimensional infrared laser scanner to measure the distance, angle, and infrared reflectivity of the surrounding environments. Given the previous pose estimate, the predicted vehicle pose, and the new measurement of laser scanner, this approach addresses the data association problem between the reflectivity of new measurement and that of the road surface in map database. Although this approach has shown to achieve centimeter-level accuracy from the driving experiments in urban roads, this approach is limited to autonomous driving due to the high cost of equipment, such as 3D laser scanner and the inertial navigation systems. It is also challenging to build/update a large-scale high-precision map database over wide geographical areas.

#### 2.2.3. Cooperative Vehicle Positioning

There have been a couple of approaches to cooperative vehicle positioning in the literature: the GPS pseudorange sharing and the crosslayer V2X positioning approaches [24,25,26,27,28,29].

The GPS pseudorange sharing approach in [24,25] cooperatively shares the GPS position fix and the propagation delay of each satellite signal, called the pseudorange. A new pseudorange-based ranging scheme is proposed in [24], where the relative distance vector to a surrounding vehicle is approximated by using the difference of their pseudoranges from the same satellite. Based on the precise road map, a cooperative map matching (CMM) scheme is presented in [25] to estimate the vehicle position by intersecting road area in the valid position area of its own GPS position fix and those from V2X communications, assuming that vehicles in the vicinity experience the same pseudorange measurement errors. The limitation of this approach is that the functionality of accessing the pseudorange of each satellite signal may not be supported by the commodity GPS devices.

The crosslayer V2X positioning approach exploits the physical-layer characteristics of received V2X communication signal, such as the Doppler-frequency shift [26], and the multilateration scheme [27,28,29], in order to estimate the relative distance to the sending vehicle. The paper in [26] presents an EKF-based tracking filter whose measurement includes the carrier frequency offset (CFO) of the received signal in order to extract the Doppler frequency shift. On the other hand, the relative distance to a neighbor vehicle in [27,28,29] is computed by the multilateration schemes, such as the received signal strength, time (difference) of arrival, and the angle of arrival. In [27], a Bayesian dithering technique is proposed to mitigate the depletion problem of particles in the particle filter based vehicle positioning. The intervehicle-communication-assisted localization (IVCAL) scheme in [28] selects the position estimate by the trilateration of three anchor vehicles supposed to have high position accuracy as the measurement input of KF, when the GPS error is high. In [29], the Bayesian maximum a posteriori (MAP) estimator is proposed to combine the GPS position fixes with the multilateration-based relative distance measurements. The benefits of the crosslayer V2X positioning approach is that there is no data association problem between the absolute and relative positioning estimates. This is because this approach exploits both physical- and application-layer information of a V2X communication signal. In practice, the multilateration scheme suffers from both the coarse grained synchronization of GPS device and the fluctuation of physical-layer parameters in time-varying multipath channels, which results in inappropriately high relative distance errors [32].

To the best of our knowledge, the ST-LRSF is the first approach to absolute vehicle positioning that associates the LSE from the ADAS radar sensor to the RSE from V2X communication. It is designed for accurate and reliable vehicle positioning against the uncertainty of GPS device, no common reference of vehicle state between them, the cardinality spanning up to a hundred of vehicles, and highly dynamic connectivity and visibility conditions. Some preliminary results have been presented in two conference papers: a greedy algorithm for sensor-data association in [30], and a spatiotemporal metric using the exponential moving average in [31]. Compared with both previous works, we make three extensions in this paper: First, this paper presents a comprehensive model for cooperative positioning which reflects the detailed limitations of automotive radars—these sensors can detect the relative radial speed using the Doppler effect, while they are oblivious to the relative tangential speed [17]. Second, it fully addresses the proposed ST-LRSF framework including the identification of vehicles in the vicinity, the derivation of covariance matrix (in Appendix A), and the detailed EKF model for ST-LRSF. Third, the impacts of a few key parameters on the positioning accuracy are demonstrated on the basis of the numerical results from the intensive packet-level simulations.

## 3. Cooperative Vehicle Positioning Model

**Figure 2:**
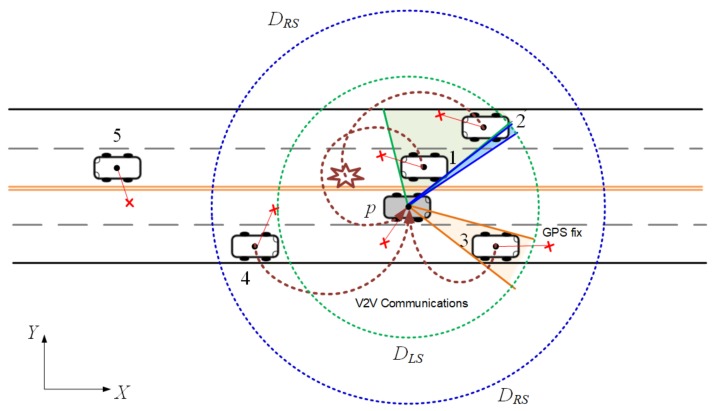
The vehicle positioning model at frame *i*.

Figure 2 shows the vehicle positioning model in a bidirectional road segment of length *L*, where each direction consists of c=2 lanes. We denote the longitudinal and the lateral directions by the *x*- and *y*-axes, respectively. We also denote the set of vehicles by V={p,1,2,3,4,5}. In this paper, each vehicle is assumed to have the following two capabilities:

1. It can estimate the relative kinematics of surrounding vehicles using its on-board radar [17], and
2. It is capable of V2X communications based on the wireless access in vehicular environments (WAVE) protocol stack [19,20].

It is clear that not every vehicle has the on-board radar at the current time. Considering the rapid growth of ADAS sensors in the marketplace, we expect that more vehicles will have the on-board radars in the near future. Without loss of generality, we focus on the positioning of reference vehicle, called the pivot vehicle *p*, as shown in Figure 2. Although the ST-LRSF framework can be used for the situation where each vehicle has its own communication and sensing ranges, we make the following two assumptions for the simplicity of expression:

1. Each vehicle has the same communication range, called the remote sensing range (RSR) $D_{RS}$.
2. Each vehicle has the same sensing range, called the local sensing range (LSR) $D_{LS}$.

Since the communication range is usually much longer than the sensing range [10,17,19], we can draw two concentric circles for each pivot vehicle, as shown in Figure 2.

### 3.1. Remote Sensing Estimate

Each vehicle is assumed to have a GPS device, a speedometer, and an orientation sensor (The GPS device can provide the speed and the heading estimates, if these sensors are not available in a vehicle). Then, all vehicles are synchronized with the GPS reference time which is partitioned into a sequence of frames with period *T*. At the start of the *i*-th frame, vehicle v∈V generates a vehicle state $X_{i}\left(v\right)$ represented by a vector in four-dimensional space, as follows:

(1) $$X_{i}\left(v\right)={\left[x_{i}\left(v\right)\;\;\;\left|{\dot{x}}_{i}\left(v\right)\right|\;\;\;\theta_{i}\left(v\right)\right]}^{⊤}={\left[x_{i}\left(v\right)\;\;\;y_{i}\left(v\right)\;\;\;\sqrt{{\dot{x}}_{i}^{2}\left(v\right)+{\dot{y}}_{i}^{2}\left(v\right)}\;\;\;\theta_{i}\left(v\right)\right]}^{⊤},$$

where $x_{i}\left(v\right)$, $|{\dot{x}}_{i}\left(v\right)|$, and $\theta_{i}\left(v\right)$ are the position, the speed, and the heading of vehicle *v*, respectively.

It is clear that the true vehicle state is unknown due to the measurement error of on-board sensors. In this paper, we assume that each type of measurement noise is mutually uncorrelated zero-mean white Gaussian. Then, the state of vehicle k∈V at frame *i* can be represented by

(2) $${\left[{\tilde{x}}_{i}\left(k\right)\;\;\;\left|{\tilde{\dot{x}}}_{i}\left(k\right)\right|\;\;\;{\tilde{\theta }}_{i}\left(k\right)\right]}^{⊤}={\left[x_{i}\left(k\right)+B+X\;\;\;\left|{\dot{x}}_{i}\left(k\right)\right|+V\;\;\;\theta_{i}\left(k\right)+\Theta \right]}^{⊤},$$

where $B={\left(B_{x},B_{y}\right)}^{⊤}$ and $X={\left(X_{x},X_{y}\right)}^{⊤}$ are the bias and the random errors of GPS device, respectively. To focus on the random GPS error, we assume that the expected differential correction E[B] is available for bias cancellation, i.e.,

(3) $$E\left[B\right]=\frac{1}{|K_{p,i}|}\sum_{k=1}^{|K_{p,i}|}\left({\tilde{x}}_{i}\left(k\right)-x_{i}\left(k\right)\right),$$

where $K_{p,i}$ is the set of neighbor vehicles at frame *i*. *V* and Θ∈[−π,π) are the random variable for speed and orientation error, respectively. We assume that the GPS error is isotropic, i.e., both $X_{x}$ and $X_{y}$ have the same STD $\sigma_{X}/\sqrt{2}$. We also denote the STD of speed and oriention noise by $\sigma_{V}$ and $\sigma_{\Theta }$, respectively.

For better awareness of neighbor vehicles in V2X communications, each vehicle periodically generates RSE $m_{i}\left(k\right)$ at the start of the *i*-th frame, as follows:

(4) $$m_{i}\left(k\right)={\left[k\;\;\;iT\;\;\;{\tilde{x}}_{i}\left(k\right)\;\;\;\left|{\tilde{\dot{x}}}_{i}\left(k\right)\right|\;\;\;{\tilde{\theta }}_{i}\left(k\right)\right]}^{⊤},$$

where *k* is the number chosen for vehicle identification in the SAE J2735 standard [21]. This message is known as the basic safety message (BSM) in US [21] and the cooperative awareness message (CAM) in Europe [22]. It is encapsulated into the beacon frame of the IEEE 802.11p MAC which will be broadcast in 5.9 GHz dedicated short-range communication (DSRC) channel at random time $t\in \left[iT,(i+1)T\right)$ [18,19,20]. Since the V2X communication is performed in unreliable DSRC channel, some neighbor vehicles may not receive the beacon. For example, in Figure 2, vehicle *p* does not receive the beacon from vehicle 2 among four vehicles in the RSR. This is due to the signal interference in the radio-propagation channel, the frame collision, or the hidden-node problems in the MAC [10,34]. Then, the set of remotely sensed vehicles (RSVs) of pivot vehicle *p* is defined as the subset of vehicles in RSR whose beacons are successfully received by pivot vehicle *p*, e.g., $K_{p,i}=\left\{1,3,4\right\}$ in Figure 2.

### 3.2. Local Sensing Estimate

The on-board radar sensors have been commercialized to fulfill the requirements of the ADAS, i.e., the estimation of the relative distance, the centrifugal speed, and the angle of a surrounding vehicle [10,17]. Based on these estimates, the systems generate alarms/warnings, or possibly control the vehicle at a hazardous situation. In this paper, we make two assumptions: (1) the on-board radar can detect the relative kinematic state of vehicles in LSR $D_{LS}$ regardless of the sensing angle, and (2) since the pivot vehicle is moving and the radar cycle is shorter than period *T*, the radar tracker can successfully distinguish the reflected signals of the targets from the clutters at random positions.

Although the relative distance is less than LSR $D_{LS}$, it is still possible that the radar may not detect the vehicle if it is not directly visible by the radar. This situation happens when another vehicle in the middle blocks the RF signal of on-board radar. Consequently, to sense surrounding vehicle *n* in the LSR, the angular width of this vehicle, denoted by $\Delta \phi_{p,i}\left(n\right)$, must be greater than the angular resolution of on-board radar, denoted by η. For example, vehicle 2 in Figure 2 is obstructed by vehicle 1, and thus cannot be detected by pivot vehicle *p*, i.e., $\Delta \phi_{p,i}\left(2\right)\leq \eta$. Then, the set of locally sensed vehicles (LSVs) is defined as the subset of vehicles in LSR that are detected by the on-board radar, e.g., $N_{p,i}=\left\{1,3\right\}$ in Figure 2.

**Figure 3:**
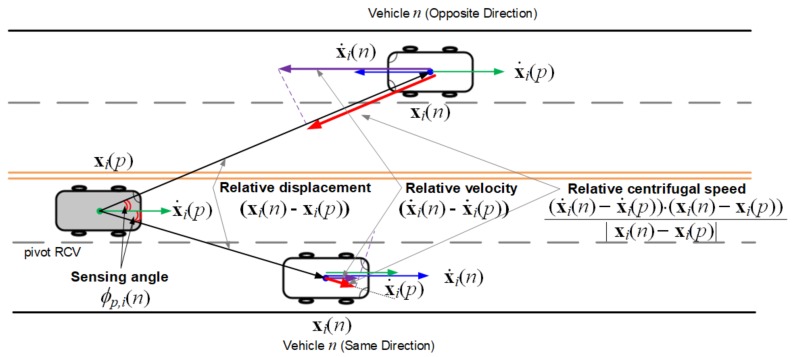
Relative kinematics of two vehicles running toward the opposite direction.

Next, we model the relative kinematic state of sensed vehicle *n*. In Figure 3, the pivot vehicle senses the LSE of two vehicles running toward the opposite direction. Denoting the relative displacement of sensed vehicle *n* from pivot vehicle *p* at frame *i* by $\Delta x_{p,i}\left(n\right)={\left[\Delta x_{p,i}\left(n\right)\;\;\;\Delta y_{p,i}\left(n\right)\right]}^{⊤}$, the relative distance $|\Delta {\hat{x}}_{p,i}\left(n\right)|$ can be estimated by

(5) $$|\Delta {\hat{x}}_{p,i}\left(n\right)|=|x_{i}\left(n\right)-x_{i}\left(p\right)|+Y,$$

where *Y* is a random variable for the measurement noise of on-board radar. We denote by $\sigma_{Y}$, the STD of distance measurement noise *Y*. The projection of relative velocity ${\dot{x}}_{i}\left(n\right)-{\dot{x}}_{i}\left(p\right)$ onto the relative displacement $x_{i}\left(n\right)-x_{i}\left(p\right)$ is defined as the relative centrifugal speed $\Delta {\dot{x}}_{p,i}^{C}\left(n\right)$, which is estimated from the Doppler frequency shift of the reflected signal [17]. We use random variable *Z* for the measurement noise of relative centrifugal speed. Denoting the STD of this noise by $\sigma_{Z}$, the estimate $\Delta {\hat{\dot{x}}}_{p,i}^{C}\left(n\right)$ of relative centrifugal speed can be expressed as follows:

(6) $$\Delta {\hat{\dot{x}}}_{p,i}^{C}\left(n\right)=\Delta {\dot{x}}_{p,i}^{C}\left(n\right)+Z=\frac{\left({\dot{x}}_{i}\left(n\right)-{\dot{x}}_{i}\left(p\right)\right)\cdot \left(x_{i}\left(n\right)-x_{i}\left(p\right)\right)}{|x_{i}\left(n\right)-x_{i}\left(p\right)|}+Z.$$

Notice that if sensed vehicle *n* approaches to pivot vehicle *p*, the relative centrifugal speed in Equation (6) becomes negative. The sensing angle $\phi_{p,i}\left(n\right)$ is also defined as the relative angle between the displacement vector $\Delta x_{p,i}\left(n\right)$ and the heading $\theta_{i}\left(p\right)$ of pivot vehicle *p*. Then, the estimate ${\hat{\phi }}_{p,i}\left(n\right)$ of sensing angle is represented by

(7) $${\hat{\phi }}_{p,i}\left(n\right)=∠\left(\Delta x_{p,i}\left(n\right)\right)-\theta_{i}\left(p\right)+\Phi ,$$

where Φ∈[−π,π) is a random variable for the angular measurement noise with STD $\sigma_{\Phi }$.

Finally, we formulate four-tuple LSE $\Delta s_{p,i}\left(n\right)$ of vehicle *n* in pivot vehicle *p* at the start of the *i*-th frame into

(8) $$\Delta s_{p,i}\left(n\right)={\left[n\;\;\;iT\;\;\;\left|\Delta {\hat{x}}_{p,i}\left(n\right)\right|\;\;\;\Delta {\hat{\dot{x}}}_{p,i}^{C}\left(n\right)\;\;\;{\hat{\phi }}_{p,i}\left(n\right)\right]}^{⊤},$$

where *n* is the unique track number of radar tracker. Since both indexes *k* and *n* are the identification numbers having no further information except for the kinematics, it is important to correctly match a pair of these indexes to improve the accuracy of vehicle positioning.

## 4. Problem Specification

Sensor fusion techniques intelligently combine multiple heterogeneous sensor data so that the resulting data have less uncertainty than the individual sensor data [35]. Since each piece of sensor data is a mixture of the ground truth and the measurement errors, there is the origin uncertainty problem in the fusion of multiple heterogeneous sensing data [13,14,33]. The existing approaches to tackling this problem are classified into two categories: the probabilistic and the distance-based approaches. The former approach addresses this problem by integrating a tracking filter with a probabilistic estimator, such as the Bayesian MAP and the maximum likelihood (ML) estimator [13,33]. This approach can obtain the optimal solution, but the complexity of computing the association probability of all valid pairs may grow exponentially with the number of targets. On the other hand, the latter approach utilizes the fact that the two sensing estimates originated from the same source will have similar values in their representation. If the metric for evaluating the dissimilarity between two points is defined well, the remaining problem is how to associate them based on the metric [14].

In this paper, we formulate our research problem based on the distance-based approach. We attempt to improve the position accuracy of pivot vehicle *p* at the end of *i*-th frame based on a new framework of sensor fusion: given its own RSE $m_{i}\left(p\right)$, the set of RSEs $\left\{m_{i}\left(k\right)|k\in K_{p,i}\right\}$ via the V2X communications, and the set of LSEs $\left\{\Delta s_{p,i}\left(n\right)|n\in N_{p,i}\right\}$ detected by its on-board radar, the objectives of sensor fusion framework are:

1. to define a spatiotemporal dissimilarity metric between two sensing estimates to reduce the instant randomness of GPS device,
2. to design the association algorithm between an element of $|K_{p,i}|$ (absolute) RSEs and an element of $|N_{p,i}|$ (relative) LSEs, i.e., k↔n, that minimizes a dissimilarity metric; and
3. to design the position refinement algorithm that reduces the impacts of dominant random GPS error X based on the LSE-RSE pairs of sensing data.

In vehicular environments, it is challenging to achieve these objectives, due to the uncertainty of GPS device [5], no common reference of vehicle state [10], the cardinality spanning up to a hundred of vehicles [36], and highly dynamic connectivity and visibility conditions [37]. Since the DSRC channel congestion can significantly deteriorate the performance of safety-critical applications [36], it is also within our interests to find the solution that achieves these challenging goals without incurring any additional messages to the V2X communications.

## 5. Spatiotemporal Local-Remote Sensor Fusion (ST-LRSF)

In this section, we present the ST-LRSF scheme that improves the position accuracy of pivot vehicle through the fusion of (absolute) RSE $\left\{m_{i}\left(k\right)|k\in K_{p,i}\right\}$ and (relative) LSE $\left\{\Delta s_{p,i}\left(n\right)|n\in N_{p,i}\right\}$. The ST-LRSF scheme first addresses the problem of identifying the vehicles in both RSR $D_{RS}$ and LSR $D_{LS}$. Next, it defines the reference vehicle state to which both RSE $m_{i}\left(k\right)$ and LSE $\Delta s_{p,i}\left(n\right)$ can be converted. It also defines the dissimilarity metric $\left\{w_{p,i}\left(k,n\right)|k\in K_{p,i},n\in N_{p,i}\right\}$ for a pair of reference vehicle states. Then, a GSA algorithm is presented to find an MWM between both reference states. Based on the outcome of MWM, the ST-LRSF scheme finally refines the position of pivot vehicle by comparing the *center of mass (CoM)* of RSE polygon with that of LSE polygon.

We also show that the refined position of ST-LRSF can be used as a measurement input of EKF model in order to further improve the accuracy of vehicle positioning.

### 5.1. Identification of Sensed/Neighbor Vehicles

At each frame *i*, the set of RSVs $K_{p,i}$ and the set of LSVs $N_{p,i}$ are constructed from the received RSEs $\left\{m_{i}\left(k\right)\right\}$ and the sensed LSEs $\left\{\Delta s_{p,i}\left(n\right)\right\}$, respectively. In general, these sets do not reflect the ground truth because of the following two factors: the lost RSEs due to the beacon loss at the DSRC channel, and the missed LSEs originating from the RF-signal obstruction. To better estimate the ground truth, the ST-LRSF scheme employs the *constant-speed estimator (CSE)* that linearly interpolates the current position of vehicles based on the latest estimate of vehicle state [36].

To distinguish the beacon loss at the DSRC channel from the neighbor vehicle out of RSR $D_{RS}$, the pivot vehicle defines the candidate set of RSVs $K_{p,i}$. On receiving RSE $m_{i}\left(k\right)$, the pivot vehicle inserts vehicle *k* to sets $K_{p,i}$ and $K_{p,i}$. At the end of frame *i*, the pivot vehicle estimates the relative distance to each vehicle $k^{\prime }\in K_{p,i-1}-K_{p,i}$ based on the CSE, i.e.,

(9) $$|\Delta {\tilde{x}}_{i}\left(p,k^{\prime }\right)\left|=\right|{\tilde{x}}_{j}\left(k^{\prime }\right)+\left(i-j\right){\tilde{\dot{x}}}_{j}\left(k^{\prime }\right)-{\tilde{x}}_{i}\left(p\right)|,$$

where *j* is the frame index in which the latest beacon of vehicle $k^{\prime }$ is received by the pivot vehicle. If the distance in Equation (9) is no greater than the RSR, the pivot vehicle interprets that the beacon of vehicle $k^{\prime }$ is lost at the DSRC channel, and thus keeps it in set $K_{p,i}$. Otherwise, it does not insert vehicle $k^{\prime }$ to set $K_{p,i}$ and discards all corresponding weights $\left\{w_{p,i}\left(k^{\prime },n\right)|n\in N_{p,i}\right\}$ because it is considered as the target out of RSR.

The pivot vehicle also utilizes the candidate set of LSVs $N_{p,i}$ to distinguish the obstruction of RF signal from the target out of LSR $D_{LS}$. For each LSE $\Delta s_{p,i}\left(n\right)$, the pivot vehicle inserts vehicle *n* to sets $N_{p,i}$ and $N_{p,i}$. At the end of frame *i*, the pivot vehicle estimates the relative distance to each vehicle $n^{\prime }\in N_{p,i-1}-N_{p,i}$ using the CSE, as follows:

(10) $$|\Delta {\hat{x}}_{p,i}\left(n^{\prime }\right)|=|\Delta {\hat{x}}_{p,j}\left(n^{\prime }\right)+\left(i-j\right)\Delta {\hat{\dot{x}}}_{p,j}\left(n^{\prime }\right)|,$$

where *j* is the index of the latest frame where vehicle $n^{\prime }$ is detected by the pivot vehicle. If the distance in Equation (10) is no greater than the LSR, the pivot vehicle infers that the radar signal to vehicle $n^{\prime }$ is obstructed by an object, and still keeps vehicle $n^{\prime }$ in set $N_{p,i}$. Otherwise, it does not insert vehicle $n^{\prime }$ to set $N_{p,i}$ and discards all corresponding weights $\left\{w_{p,i}\left(k,n^{\prime }\right)|k\in K_{p,i}\right\}$ because it is considered to be out of LSR.

### 5.2. Conversion to the Reference Vehicle State

Notice that RSE $m_{i}\left(k\right)$ in Equation (4) includes a few absolute vehicle kinematic parameters, while LSE $\Delta s_{p,i}\left(n\right)$ in Equation (8) has a few relative vehicle kinematic parameters to those of pivot vehicle. Since both of them also have random measurment errors, a unified vehicle state must be determined to match a pair of RSE $m_{i}\left(k\right)$ and LSE $\Delta s_{p,i}\left(n\right)$ based on the dissimilarity metric $w_{p,i}\left(k,n\right)$. To make a common metric either relative or absolute, the ST-LRSF scheme must incorporate the RSE of pivot vehicle $m_{i}\left(p\right)$. Furthermore, it must consider the limitation of on-board radar that it cannot estimate the relative tangential speed. Taking into account both constraints, we define the *reference state* of vehicle *v* as the absolute position in a two-dimenstional Cartesian coordinate and the absolute centrifugal speed:

(11) $$S_{p,i}\left(v\right)={\left[x_{i}{\left(v\right)}^{⊤}\;\;\;{\dot{x}}_{p,i}^{C}\left(v\right)\right]}^{⊤}.$$

**Figure 4:**
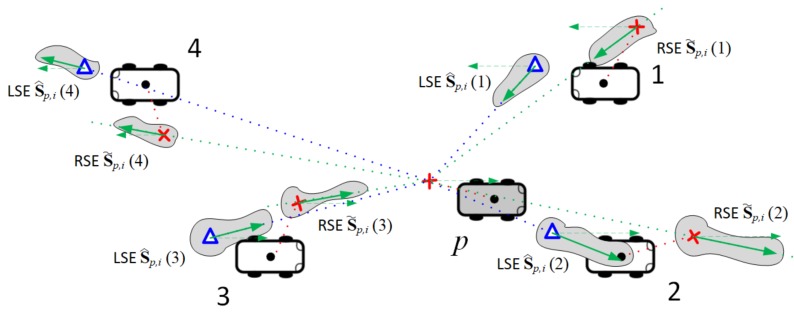
Example of reference vehicle states ${\tilde{S}}_{p,i}\left(v\right)$ and ${\hat{S}}_{p,i}\left(v\right)$ for each vehicle.

In Figure 4, each vehicle in $K_{p,i}=N_{p,i}=\left\{1,2,3,4\right\}$ has two corresponding shaded regions: one for the reference RSE ${\tilde{S}}_{p,i}\left(v\right)$ and the other for the reference LSE ${\hat{S}}_{p,i}\left(v\right)$. For $k\in K_{p,i}$, the corresponding reference RSE ${\tilde{S}}_{p,i}\left(k\right)$ is denoted by

(12) $${\tilde{S}}_{p,i}\left(k\right)={\left[{\tilde{x}}_{i}{\left(k\right)}^{⊤}\;\;\;{\tilde{\dot{x}}}_{p,i}^{C}\left(k\right)\right]}^{⊤}.$$

Since the GPS position estimate ${\tilde{x}}_{i}\left(k\right)$ marked with red cross in Figure 4 is an absolute metric, we can reuse it as the corresponding element of reference vehicle state. The absolute centrifugal speed represented by green solid arrow in each RSE region can be obtained from the projection of vehicle speed ${\tilde{\dot{x}}}_{i}\left(k\right)$ onto the sensing angle, i.e.,

(13) $${\tilde{\dot{x}}}_{p,i}^{C}\left(k\right)=\left(|{\dot{x}}_{i}\left(k\right)|+V\right)\frac{\left({\tilde{x}}_{i}\left(k\right)-{\tilde{x}}_{i}\left(p\right)\right)}{|{\tilde{x}}_{i}\left(k\right)-{\tilde{x}}_{i}\left(p\right)|}\cdot \left[\begin{array}{c} \cos{\tilde{\theta }}_{i}\left(k\right)\\ \sin{\tilde{\theta }}_{i}\left(k\right) \end{array}\right].$$

For $n\in N_{p,i}$, the ST-LRSF scheme combines the relative LSE $\Delta s_{p,i}\left(n\right)$ with the RSE of pivot vehicle $m_{i}\left(p\right)$ to obtain the reference LSE ${\hat{S}}_{p,i}\left(n\right)$:

(14) $${\hat{S}}_{p,i}\left(n\right)=\left({\hat{x}}_{p,i}\left(n\right),\;{\hat{\dot{x}}}_{p,i}^{C}\left(n\right)\right).$$

First, the absolute position estimate ${\hat{x}}_{p,i}\left(n\right)$ marked with blue triangle in Figure 4 can be represented by the sum of RSE ${\tilde{x}}_{i}\left(p\right)$ and relative distance $\Delta {\hat{x}}_{p,i}\left(n\right)$, as follows:

(15) $$\begin{array}{c} {\hat{x}}_{p,i}\left(n\right)={\tilde{x}}_{i}\left(p\right)+\left|\Delta {\hat{x}}_{p,i}\left(n\right)\right|\left[\begin{array}{c} \cos\left({\tilde{\theta }}_{i}\left(p\right)+{\hat{\phi }}_{p,i}\left(n\right)\right)\\ \sin\left({\tilde{\theta }}_{i}\left(p\right)+{\hat{\phi }}_{p,i}\left(n\right)\right) \end{array}\right]. \end{array}$$

Second, the relative centrifugal speed ${\hat{\dot{x}}}_{p,i}^{C}\left(n\right)$ depicted with the green solid arrow in each LSE region is also formulated into the projection of pivot-vehicle speed onto the sensing angle plus the relative centrifugal speed $\Delta {\hat{\dot{x}}}_{p,i}^{C}\left(n\right)$:

(16) $${\hat{\dot{x}}}_{p,i}^{C}\left(n\right)=\left|{\tilde{\dot{x}}}_{i}\left(p\right)\right|\cos\left({\hat{\phi }}_{p,i}\left(n\right)\right)+\Delta {\hat{\dot{x}}}_{p,i}^{C}\left(n\right).$$

### 5.3. The Design of Dissimilarity Metric

**Figure 5:**
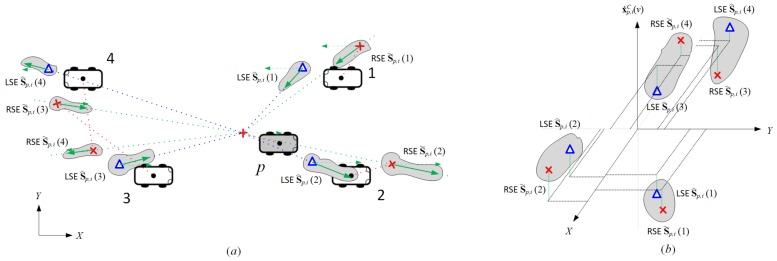
Example of mismatching between vehicles 3 and 4 based on the spatial dissimilarity in Equation (17): (**a**) example of reference vehicle states ${\tilde{S}}_{p,i}\left(v\right)$ and ${\hat{S}}_{p,i}\left(v\right)$ for each vehicle, and (**b**) the corresponding points in the Cartesian coordinate, where the origin represents the position of pivot vehicle *p* with absolute centrifugal speed of zero.

The computation of reference RSE ${\tilde{S}}_{p,i}\left(k\right)$ and reference LSE ${\hat{S}}_{p,i}\left(n\right)$ in Equations (12)–(16) involves the measurement noise of multiple different sensors possibly detecting different physical quantities, e.g., position, speed, angle, etc. Then, both estimates can be represented by a point in three-dimensional Cartesian coordinates in Figure 5b, where two axes correspond to the vehicle position and the remaining one to the absolute centrifugal speed. Since all sensor measurement errors are assumed to be zero-mean white Gaussian, the two estimates originated from the same vehicle are expected to be in close distance from each other. The goal of this section is to design a fair dissimilarity metric for given RSE ${\tilde{S}}_{p,i}\left(k\right)$ and LSE ${\hat{S}}_{p,i}\left(n\right)$. When the variance of ${\tilde{S}}_{p,i}\left(k\right)-{\hat{S}}_{p,i}\left(n\right)$ in each axis is the same, a simple Euclidean distance would be a viable solution to the dissimilarity metric. However, in general cases where the samples of ${\tilde{S}}_{p,i}\left(k\right)-{\hat{S}}_{p,i}\left(n\right)$ are spread out over each axis differently, the dissimilarity metric should reflect how many standard deviations away the LSE is from the RSE along each axis and vice versa, which is known as the Mahalanobis distance. Then, the spatial dissimilarity $d_{p,i}\left(k,n\right)$ between RSE ${\tilde{S}}_{p,i}\left(k\right)$ and LSE ${\hat{S}}_{p,i}\left(n\right)$ is represented by

(17) $$\begin{array}{c} d_{p,i}\left(k,n\right)=\sqrt{{\left({\tilde{S}}_{p,i}\left(k\right)-{\hat{S}}_{p,i}\left(n\right)\right)}^{⊤}\Sigma^{-1}\left({\tilde{S}}_{p,i}\left(k\right)-{\hat{S}}_{p,i}\left(n\right)\right)}, \end{array}$$

where Σ is the covariance matrix of ${\tilde{S}}_{p,i}\left(k\right)-{\hat{S}}_{p,i}\left(n\right)$ that is derived in the Appendix A. Since each measurement noise is an uncorrelated Gaussian, the spatial dissimilarity $d_{p,i}\left(k,n\right)$ can be approximated by the chi distribution with three degrees of freedom.

Given that pivot vehicle *p* correctly matches neighbor vehicle *k* with sensed vehicle *n* in the latest frame *j*, the corresponding weight $w_{p,j}\left(k,n\right)$ is likely to be the smallest among all contending pairs, as frame index *i* increases, i.e.,

(18) $$w_{p,j}\left(k,n\right)=\min_{\forall k^{\prime }\in K_{p,j}}w_{p,j}\left(k^{\prime },n\right)\;\mathrm{and}\;w_{p,j}\left(k,n\right)=\min_{\forall n^{\prime }\in N_{p,j}}w_{p,j}\left(n^{\prime },k\right).$$

On the other hand, if there is an mismatch in the latest frame, the corresponding weight will increase with the growth of disparity in the kinematics between neighbor vehicle *k* and sensed vehicle *n*.

Due to the instant randomness of measurement noise, the spatial dissimilarity metric results in more frequent matched pairs, as shown in Figure 5. In this example, the GPS position estimates of vehicles 3 and 4 are flipped over, which may lead to a mismatch in the GSA algorithm. To reliably match a pair of estimates against this instant randomness, the ST-LRSF scheme adopts the *spatiotemporal dissimilarity*. In this metric, weight $w_{p,i}\left(k,n\right)$ and counter $c_{p,i}\left(k,n\right)$ of matched pair $k\in K_{p,i}$ and $n\in N_{p,i}$ are computed by the cumulative moving average (CMA), as follows:

(19) $$w_{p,i}\left(k,n\right)=\frac{c_{p,j}\left(k,n\right)}{c_{p,j}\left(k,n\right)+1}w_{p,j}\left(k,n\right)+\frac{1}{c_{p,j}\left(k,n\right)+1}d_{p,i}\left(k,n\right),\;\mathrm{and}\;c_{p,i}\left(k,n\right)=c_{p,j}\left(k,n\right)+1,$$

where *j* is the latest frame index where both RSE ${\tilde{S}}_{p,i}\left(k\right)$ and LSE ${\hat{S}}_{p,i}\left(n\right)$ are available in pivot vehicle *p*.

### 5.4. A Greedy Sensor-Data Association Algorithm

In this section, we construct bipartite graph $G=(K_{p,i},N_{p,i})$ to address the association problem, where the edge connecting neighbor vehicle $k\in K_{p,i}$ and sensed vehicle $n\in N_{p,i}$ has weight $w_{p,i}\left(k,n\right)$. To reduce the computational complexity of assocation, we use gating threshold χ to filter out the assoication pairs with high spatial dissimilarity. In other words, an edge whose spatial dissimilarity in Equation (17) is no less than χ is removed in bipartite graph *G*. Then, the objective of GSA algorithm is to find a subset of edges whose vertices are all disjoint, called a matching [38]. We denote by $M_{p,i}^{*}$, the MWM consisting of $M_{p,i}=\left|M_{p,i}^{*}\right|$ edges, as follows:

(20) $$M_{p,i}^{*}=\left\{\left(k^{*}\left(u\right),n^{*}\left(u\right)\right)|k^{*}\left(u\right)\neq k^{*}\left(v\right)\;\mathrm{and}\;n^{*}\left(u\right)\neq n^{*}\left(v\right),\forall u\neq v\;\mathrm{in}\;1\leq u,v\leq M_{p,i}\right\}.$$

Notice that MWM $M_{p,i}^{*}$ is a matching having the following two characteristics:

1. $M_{p,i}^{*}$ saturates all vertices in either $K_{p,i}$ or $N_{p,i}$, or there is no edge $e\in M_{p,i}^{*}$ that connects a pair of new vertices in both sets; and
2. The sum of edge weights in $M_{p,i}^{*}$ is minimal, i.e., given $M_{p,i}^{*}$, it is not possible to further reduce the sum of weights without breaking the requirements of matching.

Algorithm 1 illustrates the pseudocode of GSA algorithm that takes bipartite graph *G* and the weight for each edge as input parameters and outputs MWM $M_{p,i}^{*}$. First, output $M_{p,i}^{*}$ and priority queue $Q_{p,i}$ are initialized to be empty (line 1). For each edge (k,n) whose spatial dissimilarity is less than χ, the GSA algorithm computes the weight of each edge, and inserts it into priority queue $Q_{p,i}$ (lines 2–9). Denoting the number of edges satisfying $d_{p,i}\left(k,n\right)<\chi$ by |E|, the running time of this operation is $O(|K_{p,i}||N_{p,i}|+|E|)$, where the first term is for computing the spatial dissimilarity and the second term is for building binary heap $Q_{p,i}$ [39]. Then, it repeats the following statements until priority queue $Q_{p,i}$ becomes empty (lines 10–16): The GSA algorithm first extracts the least-cost edge $e^{*}=\left(k^{*},n^{*}\right)$ from $Q_{p,i}$ (line 11). If neithor $k^{*}$ nor $n^{*}$ exists in the existing MWM $M_{p,i}^{*}$, the GSA algorithm inserts it into the MWM $M_{p,i}^{*}$. Otherwise, simply discards edge $e^{*}$ (lines 12–15). The running time of while loop is O(|E|log|E|). Finally, it terminates the procedure by returning $M_{p,i}^{*}$, where the overall running time of GSA algorithm is $O(|K_{p,i}||N_{p,i}|+|E|\log|E|)$ (line 17).

**Algorithm 1** Greedy Sensor-Data Association (GSA) Algorithm.**Input:** Vertex sets $K_{p,i}$ and $N_{p,i}$, and weight $w_{p,i}\left(k,n\right)$ for edge e=(k,n).**Output:** Minimal weighted matching $M_{p,i}^{*}$.1:Initialize $M_{p,i}^{*}=\phi$ and priority queue $Q_{p,i}=\phi$.2:
**for**
$k=1\to |K_{p,i}|$
**do**
3:    **for**
$n=1\to |N_{p,i}|$
**do**4:        **if**
$d_{p,i}\left(k,n\right)<\chi$
**then**5:           Compute weight $w_{p,i}\left(k,n\right)$ of e=(k,n).6:           Insert edge *e* into priority queue $Q_{p,i}$.7:        **end if**8:    **end for**9:
**end for**
10:**while**$Q_{p,i}$ is not empty **do**11:    Extract the least-cost edge $e^{*}=\left(k^{*},n^{*}\right)$ from $Q_{p,i}$.12:    **if**
$k^{*}\notin M_{p,i}^{*}$ and $n^{*}\notin M_{p,i}^{*}$
**then**13:        $M_{p,i}^{*}=M_{p,i}^{*}\cup \left\{e^{*}\right\}$.14:    **else** Discard edge $e^{*}$.15:    **end if**16:
**end while**
17:
**return**
$M_{p,i}^{*}$


We define the set of neighbor/sensed vehicles in the output of GSA algorithm $M_{p,i}^{*}$, as follows:

$$K_{p,i}^{*}=\left\{k^{*}\left(v\right)|\left(k^{*}\left(v\right),n^{*}\left(v\right)\right)\in M_{p,i}^{*}\right\}\;\mathrm{and}\;N_{p,i}^{*}=\left\{n^{*}\left(v\right)|\left(k^{*}\left(v\right),n^{*}\left(v\right)\right)\in M_{p,i}^{*}\right\},$$

where $|K_{p,i}^{*}\left|=\right|N_{p,i}^{*}|=M_{p,i}$. Assuming that $k^{*}\left(u\right)$ and $n^{*}\left(v\right)$ of MWM $M_{p,i}^{*}$ in Equation (20) indicates the same vehicle for $1\leq u,v\leq M_{p,i}$, we can partition each of these sets’ two subsets: First, the set of neighbor vehicles $K_{p,i}^{*}$ is partitioned into

$${\underline{K}}_{p,i}^{*}=\left\{k^{*}\left(u\right)|\left(k^{*}\left(u\right),n^{*}\left(u\right)\right)\in M_{p,i}^{*}\;\mathrm{and}\;\left(k^{*}\left(v\right),n^{*}\left(v\right)\right)\in M_{p,i}^{*}\right\},$$

and

$${\bar{K}}_{p,i}^{*}=\left\{k^{*}\left(u\right)|\left(k^{*}\left(u\right),n^{*}\left(u\right)\right)\in M_{p,i}^{*}\;\mathrm{and}\;\left(k^{*}\left(v\right),n^{*}\left(v\right)\right)\notin M_{p,i}^{*}\right\}.$$

Similarly, the set of sensed vehicles $N_{p,i}^{*}$ is partitioned into

$${\underline{N}}_{p,i}^{*}=\left\{n^{*}\left(v\right)|\left(k^{*}\left(v\right),n^{*}\left(v\right)\right)\in M_{p,i}^{*}\;\mathrm{and}\;\left(k^{*}\left(u\right),n^{*}\left(u\right)\right)\in M_{p,i}^{*}\right\},$$

and

$${\bar{N}}_{p,i}^{*}=\left\{n^{*}\left(v\right)|\left(k^{*}\left(v\right),n^{*}\left(v\right)\right)\in M_{p,i}^{*}\;\mathrm{and}\;\left(k^{*}\left(u\right),n^{*}\left(u\right)\right)\notin M_{p,i}^{*}\right\}.$$

### 5.5. Position Refinement by Center of Mass (PRCoM)

**Figure 6:**
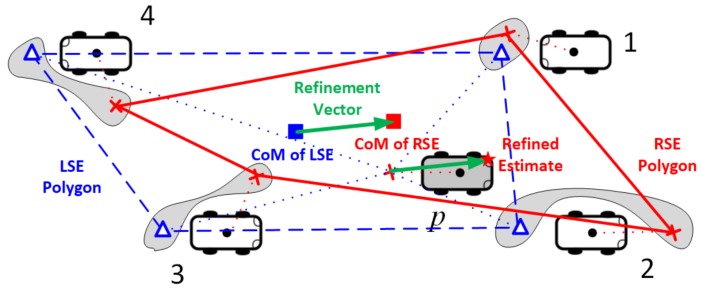
Illusration of the RSE polygon, the LSE polygon, and the PRCoM algorithm.

Given the set of matched pairs in $M_{p,i}^{*}$, we next address the design problem of the position refinement algorithm that can accurately estimate the position of pivot vehicle *p*. Figure 6 illustrates a road environment in which the pivot vehicle is running with four surrounding vehicles, each of which has a remote position estimate ${\tilde{x}}_{p,i}\left(v^{*}\right)$, marked with red cross, and a ranging measurement ${\hat{x}}_{p,i}\left(v^{*}\right)$ by the on-board radar, marked with blue triangle. The basic idea of the PRCoM is to refine the accuracy of position estimate through the comparision of the CoM of the RSE polygon, consisting of all remote position estimates ${\tilde{x}}_{i}\left(v^{*}\right)$ of $v^{*}\in K_{p,i}^{*}$, with that of LSE polygon, comprising of all local position estimates ${\hat{x}}_{p,i}\left(v^{*}\right)$ of $v^{*}\in N_{p,i}^{*}$.

The PRCoM conceptually draws the RSE polygon by visiting each position vector ${\tilde{x}}_{p,i}\left(v^{*}\right)$ at once. It also draws the LSE polygon by visiting each two-dimensional position vector ${\hat{x}}_{p,i}\left(v^{*}\right)$ at once. In Figure 6, an example of RSE polygon is represented by red solid lines, while that of LSE polygon is drawn with blue dashed lines. Notice that, regardless of visiting order, the CoM of polygon consisting of the same position vectors is the same. This is because the CoM is the average of all position vectors in the polygon. The PRCoM scheme estimates the final position of pivot vehicle by summing the self position estimate ${\tilde{x}}_{i}\left(p\right)$ and the refinement vector, which is defined as the CoM of RSE polygon subtracted by that of LSE polygon (See Figure 6).

**Theorem** **1.** In the situation where GPS error X is the dominant source of measurement noise, the RMSE of PRCoM scheme is $\sigma_{X}/\sqrt{M_{p,i}}$, given that there is no mismatching in MWM $M_{p,i}^{*}$ (${\bar{K}}_{p,i}^{*}={\bar{N}}_{p,i}^{*}=\phi$).

**Proof.**  The position error of a neighbor vehicle has no correlation with the others because the random GPS error X is *i.i.d*, as shown in Figure 6. Using Equation (2), the CoM $E({\tilde{x}}_{p,i}^{*}\left(u\right))$ of RSE polygon is formulated by

(21) $$E\left({\tilde{x}}_{p,i}^{*}\left(u\right)\right)=\frac{1}{M_{p,i}}\sum_{\forall u\in K_{p,i}^{*}}{\tilde{x}}_{i}\left(u\right)=\frac{1}{M_{p,i}}\sum_{\forall u\in K_{p,i}^{*}}\left(x_{i}\left(u\right)+X_{u}\right).$$

On the other hand, the position estimate of each sensed vehicle is the true position estimation biased by the random GPS error $X_{p}$. Since the GPS error is the dominant source of position uncertainty in Equations (15) and (A4), the CoM $E({\hat{x}}_{p,i}^{*}\left(v\right))$ of LSE polygon is formulated as follows:

(22) $$E\left({\hat{x}}_{p,i}^{*}\left(v\right)\right)=\frac{1}{M_{p,i}}\sum_{\forall v\in N_{p,i}^{*}}{\hat{x}}_{p,i}\left(v\right)\approx \frac{1}{M_{p,i}}\sum_{\forall v\in N_{p,i}^{*}}\left(x_{p,i}\left(v\right)+X_{p}\right)=\frac{1}{M_{p,i}}\sum_{\forall v\in N_{p,i}^{*}}x_{p,i}\left(v\right)+X_{p}.$$

The refinement vector ΔX of pivot vehicle is defined as the CoM of RSE polygon subtracted by that of CSE polygon. Using Equations (21) and (22), ΔX is represented by

(23) $$\Delta X=E\left({\tilde{x}}_{p,i}^{*}\left(u\right)\right)-E\left({\hat{x}}_{p,i}^{*}\left(v\right)\right)\approx \frac{1}{M_{p,i}}\left[\sum_{\forall u\in K_{p,i}^{*}}x_{i}\left(u\right)-\sum_{\forall v\in N_{p,i}^{*}}x_{i}\left(v\right)\right]+\frac{1}{M_{p,i}}\sum_{\forall u\in K_{p,i}^{*}}X_{u}-X_{p}.$$

The final estimate $x_{i}^{*}\left(p\right)$ for the position of pivot vehicle *p* can be represented by the sum of its GPS position fix ${\tilde{x}}_{i}\left(p\right)$ and the refinement vector ΔX,

(24) $$\begin{array}{cc} x_{i}^{*}\left(p\right)\; & ={\tilde{x}}_{i}\left(p\right)+\Delta X=x_{i}\left(p\right)+X_{p}+\Delta X\;\\ & \approx x_{i}\left(p\right)+\frac{1}{M_{p,i}}\left[\sum_{\forall u\in K_{p,i}^{*}}x_{i}\left(u\right)-\sum_{\forall v\in N_{p,i}^{*}}x_{i}\left(v\right)\right]+\frac{1}{M_{p,i}}\sum_{\forall u\in K_{p,i}^{*}}X_{u}. \end{array}$$

Since ${\bar{K}}_{p,i}^{*}={\bar{N}}_{p,i}^{*}=\phi$, the second term on the right-hand side (RHS) of Equation (24) cancels itself out. Then, the refined position estimate $x_{i}^{*}\left(p\right)$ can be obtained by the sum of unknown true position and the sample average of $M_{p,i}$ i.i.d zero-mean Gaussian random variables in $K_{p,i}^{*}$:

(25) $$x_{i}{\left(p\right)}^{*}\approx x_{i}\left(p\right)+\frac{1}{M_{p,i}}\sum_{\forall u\in K_{p,i}^{*}}X_{u}.$$

From the law of large numbers (LLN), the refined position estimate converges to the true position, i.e., $x_{i}^{*}\left(p\right)\to x_{i}\left(p\right)$, and the STD of final position error becomes $1/\sqrt{M_{p,i}}$ of the original GPS error X. ☐

When there exist mismatched pairs in the MWM $M_{p,i}^{*}$, the position difference between the neighbor vehicles in ${\bar{K}}_{p,i}^{*}$ and the sensed vehicles in ${\bar{N}}_{p,i}^{*}$ becomes the additional source of PRCoM positioning uncertainty, as follows:

(26) $$x_{i}^{*}\left(p\right)=x_{i}\left(p\right)+\frac{1}{M_{p,i}}\left[\sum_{\forall u\in {\bar{K}}_{p,i}^{*}}x_{i}\left(u\right)-\sum_{\forall v\in {\bar{N}}_{p,i}^{*}}x_{i}\left(v\right)\right]+\frac{1}{M_{p,i}}\sum_{\forall u\in K_{p,i}^{*}}X_{u}.$$

### 5.6. Extended Kalman Filter Model with ST-LRSF

In this section, we design the EKF model whose state $X_{i}$ is defined as that in Equation (1) (In this section, we omit the index of pivot vehicle *p* between parenthesis in all notations for the expressional simplicity.): $X_{i}={\left[\begin{array}{cccc} x_{i} & y_{i} & |{\dot{x}}_{i}| & \theta_{i} \end{array}\right]}^{⊤}$. In this model, the refined position of PRCoM scheme in Equation (24) is used as a part of measurement $Z_{i}$, i.e., $Z_{i}={\left[\begin{array}{cccc} x_{i}^{*} & y_{i}^{*} & |{\tilde{\dot{x}}}_{i}| & {\tilde{\theta }}_{i} \end{array}\right]}^{⊤}$.

The trace of vehicle mobility simulator in this paper consists of the true position $x_{i}$ and speed $|{\dot{x}}_{i}|$ [40]. To simultaneously match both position and speed of pivot vehicle in two consecutive frames, we consider a linear-acceleration mobility model whose acceleration at time t∈[(i−1)T,iT) is represented by $a_{i-1}\left(t\right)=\alpha_{i-1}t+\beta_{i-1}$, where

$$\begin{array}{c} \alpha_{i-1}=\frac{2}{T^{3}}\left[3T(|{\dot{x}}_{i}\left|-\right|{\dot{x}}_{i-1}|)-6(x_{i}-x_{i-1}-T|{\dot{x}}_{i-1}\left|\right)\right],\;\mathrm{and}\\ \beta_{i-1}=-\frac{2}{T^{3}}\left[3T(x_{i}-x_{i-1}+T|{\dot{x}}_{i-1}\left|\right)+T^{2}\left(\right|{\dot{x}}_{i}\left|-\right|{\dot{x}}_{i-1}\left|\right)\right]. \end{array}$$

In a straight road, the predicted state estimate ${\hat{X}}_{i|i-1}$ is formulated by a nonlinear differentiable function f(·), i.e.,

(27) $${\hat{X}}_{i|i-1}=f\left({\hat{X}}_{i-1|i-1},u_{i-1}\right)=\left[\begin{array}{c} {\hat{x}}_{i-1|i-1}+\left(T|\hat{\dot{x}}{|}_{i-1|i-1}+\frac{T^{2}}{2!}\beta_{i-1}+\frac{T^{3}}{3!}\alpha_{i-1}\right)\cos{\hat{\theta }}_{i-1|i-1}\\ {\hat{y}}_{i-1|i-1}+\left(T|\hat{\dot{x}}{|}_{i-1|i-1}+\frac{T^{2}}{2!}\beta_{i-1}+\frac{T^{3}}{3!}\alpha_{i-1}\right)\sin{\hat{\theta }}_{i-1|i-1}\\ |\hat{\dot{x}}{|}_{i-1|i-1}+T\beta_{i-1}+\frac{T^{2}}{2!}\alpha_{i-1}\\ {\hat{\theta }}_{i-1|i-1} \end{array}\right],$$

where ${\hat{X}}_{i-1|i-1}$ is the updated estimate of EKF at the previous frame, and $u_{i-1}$ is the control input of vehicle $u_{i-1}={\left[\begin{array}{cc} \alpha_{i-1} & \beta_{i-1} \end{array}\right]}^{⊤}$. The predicted covariance estimate $P_{i|i-1}$ is also represented by $P_{i|i-1}=F_{i-1}P_{i-1|i-1}F_{i-1}^{⊤}+Q_{i-1}$, where

$$F_{i-1}=\frac{\partial f({\hat{X}}_{i-1|i-1},u_{i-1})}{\partial {\hat{X}}_{i-1|i-1}}=\left[\begin{array}{cccc} 1 & 0 & T\cos{\hat{\theta }}_{i-1|i-1} & -\left(T|\hat{\dot{x}}{|}_{i-1|i-1}+\frac{T^{2}}{2!}\beta_{i-1}+\frac{T^{3}}{3!}\alpha_{i-1}\right)\sin{\hat{\theta }}_{i-1|i-1}\\ 0 & 1 & T\sin{\hat{\theta }}_{i-1|i-1} & \left(T|\hat{\dot{x}}{|}_{i-1|i-1}+\frac{T^{2}}{2!}\beta_{i-1}+\frac{T^{3}}{3!}\alpha_{i-1}\right)\cos{\hat{\theta }}_{i-1|i-1}\\ 0 & 0 & 1 & 0\\ 0 & 0 & 0 & 1 \end{array}\right]$$

is the Jacobian of function f(·), and noise covariance matrix $Q_{i-1}$ is set to zero.

Given the measurement $Z_{i}$ and the predicted estimate of state ${\hat{X}}_{i|i-1}$ and its covariance $P_{i|i-1}$, the expression of optimal Kalman gain $G_{i}$, the update equations for the state ${\hat{X}}_{i|i}$ and its covariance $P_{i|i}$ are identical to those of (linear) Kalman filter, as follows:

(28) $$\begin{array}{c} G_{i}=P_{i|i-1}H_{i}^{⊤}{\left(H_{i}P_{i|i-1}H_{i}^{⊤}+R_{i}\right)}^{-1},\\ {\hat{X}}_{i|i}={\hat{X}}_{i|i-1}+G_{i}\left(Z_{i}-H_{i}{\hat{X}}_{i|i-1}\right),\\ P_{i|i}=P_{i|i-1}-G_{i}H_{i}P_{i|i-1}, \end{array}$$

where $H_{i}$ is the identity matrix $I_{4}$, and measurement covariance matrix $R_{i}$ is

$$R_{i}=\left[\begin{array}{cccc} \frac{\sigma_{X}^{2}}{2M_{p,i}} & 0 & 0 & 0\\ 0 & \frac{\sigma_{X}^{2}}{2M_{p,i}} & 0 & 0\\ 0 & 0 & \sigma_{V}^{2} & 0\\ 0 & 0 & 0 & \sigma_{\Theta }^{2} \end{array}\right].$$

## 6. Numerical Results

In this section, we present the numerical results of three vehicle positioning schemes obtained from extensive simulations: the raw GPS, marked "GPS", the LRSF using spatial weight in Equation (17) only, marked "S-LRSF", and the LRSF with spatiotemporal weight in Equation (19), marked "ST-LRSF". We also compare the LRSF schemes with two ideal positioning schemes:

1. To identify the best achievable performance of LRSF scheme, we present the numerical results of LRSF with perfect matching, marked with “LRSF-PM”, by which the correct pairs of both estimates are always found for given $K_{p,i}$ and $N_{p,i}$.
2. We also compare the numerical results of LRSF schemes with those of the ideal IVCAL scheme, marked with “IVCAL” [28]. In this scheme, we assume that the system can always make the best choice between the GPS position estimate and the one by the trilateration of three position anchors having the best positioning accuracy. In other words, the position estimate closer to the ground truth is always used for the measurement input of KF.

We sequentially conduct three simulations for collecting the numerical results of these schemes. The Simulation of Urban MObility (SUMO) is used for the trace of all vehicles based on the car-following model [40]. At each time of trace, the vehicle sensing simulator (VSS) constructs the set of sensed vehicles $N_{p,i}$ for each pivot vehicle *p* using the algorithm in Appendix B. Taking this trace and the set of sensed vehicles as inputs, a detailed packet-level network simulation is done using the QualNet simulator [41].

Two vehicle mobility models are considered in a straight road segment: a ten-vehicle mobility (TVM) and a large-scale mobility (LSM). In the TVM, a group of five vehicles enters at each end of road segment, and runs toward the opposite end. On the other hand, in the LSM, a large-scale vehicle trace is produced to examine the impacts of key parameters on the positioning accuracy of LRSF schemes. The LSM simulation were carried out until $10^{5}$ positioning samples were gathered for each point of a graph. To minimize the boundary effects in the LSM, we also exclude the numerical results of vehicles in 500-m distance from both ends of road segment.

The list of simulation parameters is summarized in Table 2. In the QualNet simulation, the IEEE 802.11a physical- and MAC-layer parameters are modified to be compatible with IEEE 802.11p standard [19]. The DSRC channel is modelled by the Nakagami fading channel using the parameters from the Highway 101 bay area [34]. The association gate χ corresponds to the 99-percentile of chi distribution with three degrees of freedom in Equation (17). Table 3 also lists the STDs of RSE/LSE measurement noise in the simulation. In this table, the standard deviations of proprioceptive sensors are taken from those of sensor equipments in Table II of paper [42], while the standard deviations of exteroceptive sensors are the generic parameters of 77-GHz long-range radars in Table IV of paper [17].

### 6.1. Ten-Vehicle Mobility Model

We first investigate the performance of LRSF schemes obtained from a randomly chosen vehicle in the TVM. In this section, we also present the estimation accuracy of EKF models in Section 5.6, marked “EKF-GPS”, “EKF-IVCAL”, “EKF-S-LRSF”, “EKF-ST-LRSF”, and “EKF-LRSF-PM”.

**Figure 7:**
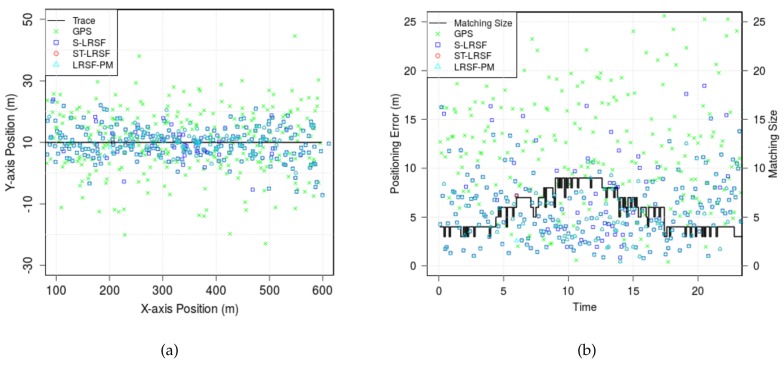
The position estimate and the positioning error of a vehicle. (**a**) vehicle position estimates when $\sigma_{X}=15$ m; (**b**) vehicle positioning error vs. time.

Figure 7 shows the estimates of positioning schemes, when T=0.1 s, $D_{LS}=200$ m, $\sigma_{X}=15$ m, and $E(|{\tilde{\dot{x}}}_{i}\left(v\right)|)=20$ m/sec. In Figure 7a, the trajectory of vehicle is shown in thick black curve, while the position estimates are represented by the marks. It is observed that the position estimates of raw GPS have the highest deviation from the trajectory, while those of three LRSF schemes are relatively close to the trajectory. Figure 7b shows a solid staircase line for the matching size $M_{p,i}$ and the markers for the positioning errors, $|X_{p}|$ and $|X^{*}\left|=\right|x_{i}\left(p\right)-x_{i}^{*}\left(p\right)|$, at each frame. We notice that the matching size is initially four, gradually increases up to nine when two vehicle groups are within each other’s sensing range, and finally decreases to four. The occasional drops in the matching size origniate from the obstruction of vehicle sensing and/or the beacon loss in the DSRC channel. We observe that both ST-LRSF and LRSF-PM schemes show the best positioning accuracy among the positioning schemes.

To evaluate the performance of the GSA algorithm, we define the probability of correct matching (PCM) as the probability that there is no mismatched entry in the MWM $M_{p,i}^{*}$, i.e., $\mathrm{Pr}\left({\bar{K}}_{p,i}^{*}={\bar{N}}_{p,i}^{*}=\phi \right)$. We notice that the ST-LRSF scheme achieves much higher PCM than the S-LRSF scheme: on average, 0.964 for the former, and 0.799 for the latter. From this result, we validate that the temporal CMA of ST-LRSF scheme successfully mitigates the instant randomness of spatial GPS errors.

**Figure 8:**
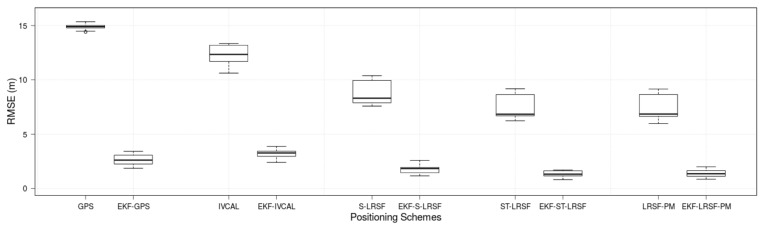
Boxplots for the RMSE of positioning schemes.

Figure 8 shows the boxplots for the RMSE of the positioning schemes and their corresponding EKF models in the TVM, where the bottom, the middle, and the top horizontal lines of each box correspond to the 25 percentile, the median, and the 75 percentile of distribution, respectively. The whiskers and the circle mark of each scheme stand for the 99 percentiles and the maximum/minimum values from the simulation results, respectively. We make several observations in this figure: First, the experimental RMSE of raw GPS is 14.9 m, which closely matches with parameter $\sigma_{X}=15.0$ m. Second, the RMSE of optimal IVCAL scheme (12.2 m) is not much better than GPS RMSE because the relative distance estimate based on the received signal strength indicator (RSSI) tends to be highly inaccurate in the Nakagami fading channel [32,34]. On the other hand, the PRCoM of LRSF schemes can achieve much lower RMSE compared to the raw GPS errors: 8.83 m, 7.49 m, and 7.44 m for the S-LRSF, ST-LRSF, and LRSF-PM schemes, respectively. Third, the EKF models can significantly reduce the RMSE of positioning schemes: on average, 82.0%, 79.8%, 82.2%, and 81.5% reduction for the GPS, S-LRSF, ST-LRSF, and LRSF-PM schemes, respectively. Surprisingly, the RMSE of EKF-IVCAL scheme is 19.9% higher than the EKF-GPS RMSE. This is because the position estimate of optimal IVCAL scheme tends to be more biased than the GPS scheme—the average bias error of the former is 1.67 m, while that of the latter is 0.846 m. For comparison, the average bias errors of S-LRSF, ST-LRSF, and LRSF-PM schemes are 0.913 m, 0.475 m, and 0.443 m, respectively. Finally, the EKF-ST-LRSF scheme achieves the least RMSE (1.34 m), which is approximately 50.0% of EKF-GPS RMSE.

### 6.2. Large-Scale Mobility Model

In this section, we examine the impacts of five parameters on the positioning accuracy of LRSF schemes: STD of raw GPS error, vehicle density, sensing range, sensing period, and vehicle speed. If not otherwise mentioned, the default settings of parameters are T=0.5 s, $D_{LS}=200$ m, $\sigma_{X}=15$ m, $E(|{\tilde{\dot{x}}}_{i}\left(v\right)|)=20$ m/s, and vehicle density of each direction ρ=90 veh/km.

#### 6.2.1. Standard Deviation of GPS Error

**Figure 9:**
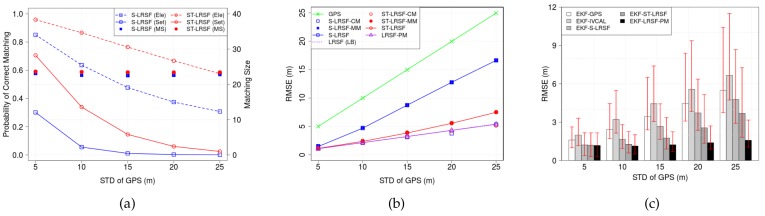
Impacts of random GPS error $\sigma_{X}$ on the accuracy of vehicle positioning. (**a**) PCM and matching size; (**b**) RMSE of LRSF schemes; (**c**) RMSE of EKF-LRSF schemes.

Figure 9 shows (a) the PCM and matching size $M_{p,i}$, (b) the RMSE of LRSF schemes, and (c) the RMSE of EKT-LRSF schemes, when the STD $\sigma_{X}$ of GPS error ranges from 5 m to 25 m. For comparison, the element-wise PCM of $\left(k^{*}\left(m\right),n^{*}\left(m\right)\right)\in M_{p,i}^{*}$ is also shown by the dashed lines in Figure 9a. For given vehicle density ρ=90 veh/km and LSR $D_{LS}=200$ m, we observe that the matching size is almost the same regardless of the spatial GPS error. On the contrary, both element- and set-wise PCMs decrease with the GPS error. This is because the increased GPS uncertainty incurs more frequent mismatchings, as shown in Figure 5. We also observe that the ST-LRSF scheme achieves higher PCM than the S-LRSF scheme, which validates that the spatiotemporal dissimilarity metric can effectively mitigate the impacts of instant GPS randomness.

In Figure 9b, we show the RMSE of three LRSF schemes. For comparison, we also plot the RMSE of correct matching (CM) $M_{p,i}^{*}\in \left\{M_{p,i}^{*}|{\bar{K}}_{p,i}^{*}={\bar{N}}_{p,i}^{*}=\phi \right\}$ with hollow marks, mismatching (MM) $M_{p,i}^{*}\in \left\{M_{p,i}^{*}|{\bar{K}}_{p,i}^{*}\neq \phi \;and\;{\bar{N}}_{p,i}^{*}\neq \phi \right\}$ with filled marks, and theoretical LB $\sigma_{X}/\sqrt{M_{p,i}}$ with dotted lines. We make four observations: First, the RMSE of raw GPS is the highest almost the same as $\sigma_{X}$. Second, the RMSE of ST-LRSF scheme is the lowest and no greater than 25.9% of raw GPS RMSE. Third, the RMSEs of S-LRSF-CM, ST-LRSF-CM, and LRSF-PM are almost the same as the RMSE of theoretical LB which is determined by the cardinality of MWM $M_{p,i}$, while the RMSE of S-LRSF-MM is much higher than that of ST-LRSF-MM. We can infer that the spatiotemporal dissimilarity metric not only increases the PCM but also decreases the impacts of mismatching term in Equation (26). Fourth, the RMSE of each LRSF scheme can be seen as the weighted RMSE sum of CM and MM: If the PCM is high, all RMSEs are close to the RMSE of theoretical LB, but the RMSE of each LRSF scheme quickly deviates from the RMSE of theoretical LB due to the fast decrease of the PCM.

Figure 9c shows the RMSEs of EKF-GPS, EKF-S-LRSF, EKF-ST-LRSF, and EKF-LRSF-PM schemes, where the bar corresponds to the average RMSE, and the bottom and the top of red whisker represent the 5 percentile and the 95 percentile of RMSE, respectively. We observe that the RMSE of EKF-GPS scheme is proportional to the GPS error, while that of EKF-LRSF-PM scheme is almost the same thanks to the PRCoM scheme. We also notice that, as the GPS error increases, the ratio of EKF-ST-LRSF RMSE to EKF-LRSF-PM RMSE in Figure 9c becomes higher than that of ST-LRSF RMSE to LRSF-PM RMSE in Figure 9b. This is because the EKF model in Section 5.6 does not take into account the error from mismatched entries in Equation (26). As a result, the Kalman gain $G_{i}$ in Equation (28) is not optimal for both S-LRSF and ST-LRSF schemes when the PCM is low. However, we still observe that the RMSE of EKF-ST-LRSF scheme is 54.3% higher than the EKF-LRSF-PM RMSE on average, but is 60.4% of EKF-GPS RMSE and 77.2% of EKF-S-LRSF RMSE.

#### 6.2.2. Vehicle Density

**Figure 10:**
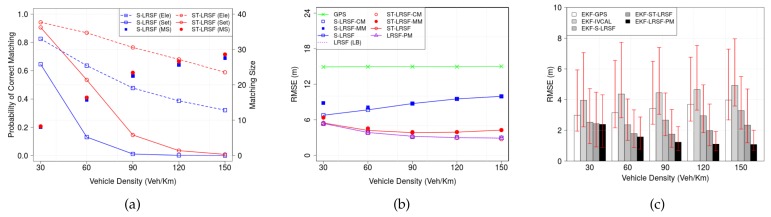
Impacts of vehicle density ρ on the accuracy of vehicle positioning. (**a**) PCM and matching size; (**b**) RMSE of LRSF schemes; (**c**) RMSE of EKF-LRSF schemes.

Figure 10 shows (a) the PCM and matching size, (b) the RMSE of LRSF schemes, and (c) the RMSE of EKT-LRSF schemes, when vehicle density ρ ranges from 30 veh/Km to 150 veh/Km. In Figure 10a, the matching size increases sublinearly with vehicle density due to the obstruction. From the LLN, the RMSE of theoretical LB is inversely proportional to the square root of matching size, as shown in Figure 10b. On the contrary, the RMSE of raw GPS can be seen as a horizontal line, because the position uncertainty of standalone GPS device is independent of vehicle density. We also make similar observations: when the PCM is high, the RMSE of each LRSF scheme is close to that of correct matching, but it approaches the RMSE of mismatching as the PCM decreases with vehicle density. Since the inter-vehicle distance is inversely proportional to vehicle density, the increase of vehicle density results in more frequent mismatchings. Although the RMSE of ST-LRSF scheme is the lowest and close to the RMSE of theoretical LB, the RMSE of EKF-ST-LRSF scheme also deviates from that of EKF-LRSF-PM scheme at high vehicle density whose PCM is close to zero. Finally, the average EKF-ST-LRSF RMSE 50.9% higher than the EKF-LRSF-PM RMSE, but is 60.4% of EKF-GPS RMSE and 75.3% of EKF-S-LRSF RMSE.

#### 6.2.3. Sensing Range

**Figure 11:**
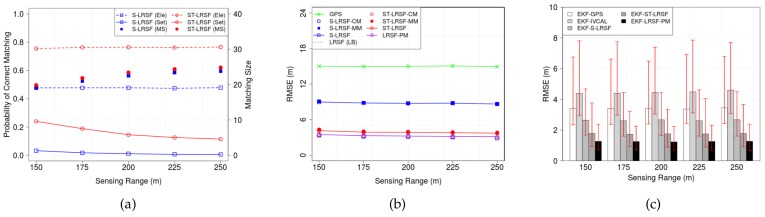
Impacts of LSR $D_{LS}$ on the accuracy of vehicle positioning. (**a**) PCM and matching size; (**b**) RMSE of LRSF schemes; (**c**) RMSE of EKF-LRSF schemes.

Figure 11 shows (a) the PCM and matching size, (b) the RMSE of LRSF schemes, and (c) the RMSE of EKT-LRSF schemes, when LSR $D_{LS}$ ranges from 150 m to 250 m. In Figure 11a, we see that the matching size increases with LSR, which slightly reduces the RMSE of corresponding LBs in Figure 11b. We also observe that the set-wise PCM slightly decreases with LSR, which may result in the increase of RMSE in Figure 11b. Among these two conflicting factors, the RMSE in Figure 11b shows that the former seems to be more significant than the latter. We also notice that the ST-LRSF scheme achieves the lowest RMSE, which is almost close to that of LRST-PM scheme. Finally, the EKF-ST-LRSF RMSE is 55.0% higher than the EKF-LRSF-PM RMSE, but is 51.5% of EKF-GPS RMSE and 66.6% of EKF-S-LRSF RMSE.

#### 6.2.4. Sensing Period

**Figure 12:**
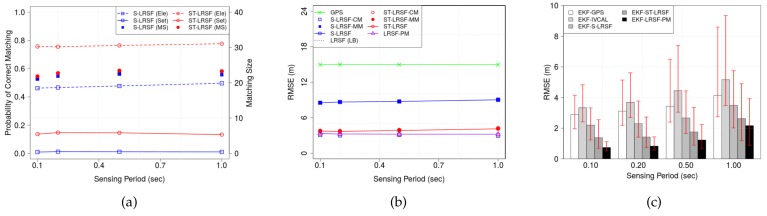
Impacts of sensing period *T* on the accuracy of vehicle positioning. (**a**) PCM and matching size; (**b**) RMSE of LRSF schemes; (**c**) RMSE of EKF-LRSF schemes.

Figure 12 shows (a) the PCM and matching size, (b) the RMSE of LRSF schemes, and (c) the RMSE of EKT-LRSF schemes, when sensing period *T* ranges from 0.1 s to 1.0 s. In Figure 12a, we observe that the matching size slightly increases with sensing period because of the reduced DSRC channel congestion, which results in less number of lost beacons. This phenomenon leads to a slight increase in the element-wise PCM of both LRSF schemes. In Figure 12b, the RMSE of each LRSF scheme slightly increases with sensing period, which means that the impact of increased matching size is almost negligible. We also make three observations: first, both the raw GPS error and the LBs have a constant value because they are independent of sensing period; second, the RMSE of ST-LRSF scheme is the least and close to the corresponding LB on average. Finally, the EKF-ST-LRSF RMSE is 39.9% higher than the EKF-LRSF-PM RMSE but is about 52.0% of EKF-GPS RMSE and 66.4% of EKF-S-LRSF RMSE.

#### 6.2.5. Vehicle Speed

**Figure 13:**
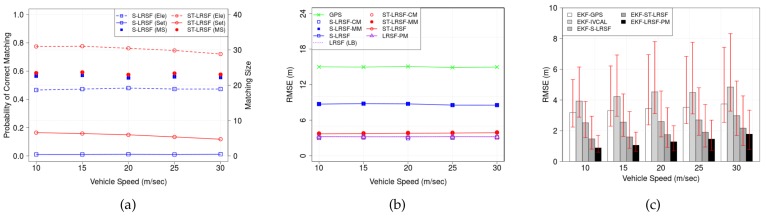
Impacts of vehicle speed $E(|{\tilde{\dot{x}}}_{i}\left(v\right)|)$ on the accuracy of vehicle positioning. (**a**) PCM and matching size; (**b**) RMSE of LRSF schemes; (**c**) RMSE of EKF-LRSF schemes.

Figure 13 shows (a) the PCM and matching size, (b) the RMSE of LRSF schemes, and (c) the RMSE of EKT-LRSF schemes, when vehicle speed $E(|{\tilde{\dot{x}}}_{i}\left(v\right)|)$ ranges from 10 m/s to 30 m/s. In Figure 13a, the PCM of ST-LRSF scheme slightly decreases with vehicle speed due to the reduced temporal correlation originating from vehicle dynamics, while that of S-LRSF scheme is almost a constant. In addition, a high vehicle speed may increase multipath fading, which results in a slight decrease of matching size. The mixture of above factors leads to the opposite trend in both LRSF schemes: The RMSE of ST-LRSF scheme slightly increases with vehicle speed, while that of S-LRSF scheme slightly decreases in Figure 13b. We also observe that the EKF-ST-LRSF RMSE is 41.2% higher than the EKF-LRSF-PM RMSE, but is 51.5% of EKF-GPS RMSE and 66.3% of EKF-S-LRSF RMSE.

From all aforementioned results, we conclude that both the basic and the EKF ST-LRSF schemes achieve the best RMSE performance for a wide range of parameters, when compared with the corresponding GPS, IVCAL, and S-LRSF schemes.

## 7. Conclusions

In this paper, we present the framework of spatiotemporal local-remote sensor fusion scheme that can reduce the random GPS error by comparing the relative state measured at the on-board radar with the absolute state received from neighbor vehicles. The ST-LRSF scheme formulates the state association problem between both estimates of vehicle state into the MWM problem in a bipartite graph, where the edge cost represents the spatiotemporal dissimilarity. Based on the MWM, we also present the PRCoM scheme, where the resulting position uncertainty is reduced by aggregating the random GPS errors of all vehicles in the MWM. Assuming that every matched entry in the MWM is a correct matching, we prove that the position accuracy is improved by the square root of matching size. We also present an EKF model, where the output of PRCoM scheme is used as the measurement input of EKF. Numerical results show that the ST-LRSF scheme can achieve the best positioning performance, regardless of raw GPS error, vehicle density, LSR, sensing period, and vehicle speed.

A generalization of the ST-LRSF framework to the positioning reference of a real vehicle may require a few directions for future work. First, considering the current penetration ratio of the automotive radars, further research is necessary to design an extended ST-LRSF scheme by which a vehicle without radar can improve its position accuracy based on the sharing of LSEs through V2X communications. Since the bias error in the position estimate may degrade the performance of (E)KF, an efficient technique for GPS bias cancellation in Equation (3) has to be studied as further work. Next, this paper presents a simple GSA algorithm for the matching between LSE and RSE, but the numerical results show that an improved sensor-data association algorithm may additionally reduce the RMSE of EKF-ST-LRSF scheme approximately up to 50%. Finally, this work proposes the PRCoM scheme to reduce the RMSE of pivot vehicle based on the assumption that the GPS error of each vehicle has the same STD, but a new positioning refinement model relaxing this assumption should also be investigated as further work.

## Figures and Tables

**Table 1:** Summary of vehicle positioning approaches.

Approach	Research	PositionReference	Tracking	RelativeSensing	V2XComm.	Map Matching
Relative Vehicle Positioning	Bar-Shalom et al. [13]	-	(J)PDAF	Radar	-	-
	Müller et al. [33]	-	UKF	Radar	Beacon	-
	Yuan et al. [14]	GPS	IMM	Radar	Beacon	-
Standalone Vehicle Positioning	Abbott et al. [7]	(D)GPS	KF	-	-	-
	Cui et al. [8]	GPS	EKF + IMM	-	-	-
	Rezaei et al. [9]	DGPS	EKF + bicycle model	-	-	-
Map-based Vehicle Positioning	Levinson et al. [15]	GPS+IMU	Particle Filter	3D laser scanner	-	SLAM
	Levinson et al. [16]	GPS+IMU	Bayesian Inference	3D laser scanner	-	SLAM
Cooperative Vehicle Positioning	Liu et al. [24]	GPS	-	Pseudorange Difference	Beacon + RDVs	-
	Rohani et al. [25]	GPS	-	-	Pseudorange Correction	CMM-PF
	Alam et al. [26]	GPS	EKF	CFO	Beacon	-
	Hoang et al. [27]	GPS	Particle Filter	IR-UWB ToA	Beacon	-
	Drawil et al. [28]	GPS	KF	multi-lateration	Beacon	-
	Rohani et al. [29]	-	Bayesian MAP	multi-lateration	Beacon	-
	Our Work	DGPS	EKF	Radar	Beacon	-

CMM-PF: CMM with Particle Filter; IMU: Inertial Measurement Unit; IR-UWB: Impulse Radio Ultra-Wideband; KF: Kalman Filter; RDV: Relative Distance Vector.

**Table 2:** List of simulation parameters.

Parameter	Notation	Value	Unit
TVM road length	$L_{TVM}$	600	m
LSM road length	$L_{LSM}$	6000	m
Number of lanes	*c*	4	-
Lane width	-	4	m
Vehicle length	-	4	m
Vehicle width	-	2	m
Data rate	*r*	6	Mbps
beacon length	*l*	300	bytes
beacon transmit power	*P*	20	dBm
Angular resolution	η	0.5	deg
Association gate	χ	3.3675	-

**Table 3:** Standard deviation of measurement noise.

Measurement Noise	Notation	Value	Unit
Vehicle speed	$\sigma_{V}$	0.3	m/s
Vehicle heading	$\sigma_{\Theta }$	0.5	deg
Relative distance	$\sigma_{Y}$	0.1	m
Relative centrifugal speed	$\sigma_{Z}$	0.1	m/s
Sensing angle	$\sigma_{\Phi }$	0.1	deg

**Figure A1:**
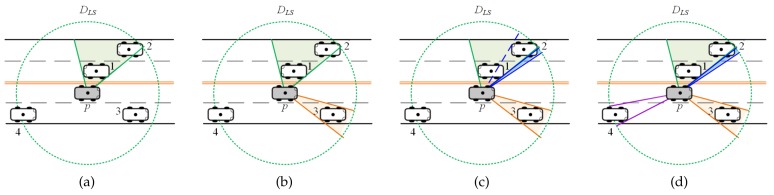
Simulation of exteroceptive sensing. (**a**) Step 1: $N_{p,i}=\left\{1\right\}$; (**b**) Step 2: $N_{p,i}=\left\{1,3\right\}$; (**c**) Step 3: $N_{p,i}=\left\{1,3\right\}$; (**d**) Step 4: $N_{p,i}=\left\{1,3,4\right\}$.

## Appendix A. The Covariance Matrix of Spatial Dissimilarity

The covariance matrix Σ in Equation (17) can be represented by

(A1) $$\begin{array}{c} \Sigma =\left[\begin{array}{ccc} \sigma_{1}^{2} & \sigma_{1,2}^{2} & \sigma_{1,3}^{2}\\ \sigma_{2,1}^{2} & \sigma_{2}^{2} & \sigma_{2,3}^{2}\\ \sigma_{3,1}^{2} & \sigma_{3,2}^{2} & \sigma_{3}^{2} \end{array}\right], \end{array}$$

where each diagonal entry is the variance of each element in ${\tilde{S}}_{p,i}\left(k\right)-{\hat{S}}_{p,i}\left(n\right)$, and $\sigma_{u,v}^{2}\;\left(u\neq v\right)$ is the covariance between the *u*-th and the *v*-th element of ${\tilde{S}}_{p,i}\left(k\right)-{\hat{S}}_{p,i}\left(n\right)$. Notice that the covariance matrix Σ is a symmetric matrix, i.e., $\sigma_{u,v}^{2}=\sigma_{v,u}^{2}$. In this Appendix, we attempt to derive the approximate equation for each element of covariance matrix Σ.

Based on Equations (2), (5), and (15), the random position difference ${\tilde{x}}_{i}\left(k\right)-{\hat{x}}_{p,i}\left(n\right)$ can be represented by

(A2) $$\begin{array}{c} \begin{array}{cc} {\tilde{x}}_{i}\left(k\right)-{\hat{x}}_{p,i}\left(n\right)\; & =x_{i}\left(k\right)+X_{k}-x_{i}\left(p\right)-X_{p}-\left(|x_{i}\left(n\right)-x_{i}\left(p\right)|+Y\right)\left[\begin{array}{c} \cos(A+B)\\ \sin(A+B) \end{array}\right], \end{array} \end{array}$$

where $X_{k}$ and $X_{p}$ are the random GPS errors of vehicle *k* and *p*, respectively. *A* is the true absolute sensing angle of vehicle *n*, i.e., $A=\theta_{i}\left(p\right)+\phi_{p,i}\left(n\right)$, and *B* is the sum of two zero-mean angular random variables, i.e., $B=\Theta_{p}+\Phi_{n}$. Assuming that cosB≈1 and sinB≈B, we can approximate the following sub-equation in Equation (A2), as follows:

(A3) $$\begin{array}{c} \begin{array}{c} x_{i}\left(k\right)-x_{i}\left(p\right)-\left|x_{i}\left(n\right)-x_{i}\left(p\right)\right|\left[\begin{array}{c} \cos(A+B)\\ \sin(A+B) \end{array}\right]\\ \;\;\;\;\approx \left(x_{i}\left(k\right)-x_{i}\left(n\right)\right)+\left(x_{i}\left(n\right)-x_{i}\left(p\right)\right)-\left|x_{i}\left(n\right)-x_{i}\left(p\right)\right|\left[\begin{array}{c} \cos A-B\sin A\\ \sin A+B\cos A \end{array}\right]\\ \;\;\;\;\approx x_{i}\left(k\right)-x_{i}\left(n\right)-B\left|\Delta x_{p,i}\left(n\right)\right|\left[\begin{array}{c} -\sin A\\ \cos A \end{array}\right]. \end{array} \end{array}$$

Substituting Equation (A3) into Equation (A2), we have

(A4) $$\begin{array}{cc} \;{\tilde{x}}_{i}\left(k\right)-{\hat{x}}_{p,i}\left(n\right)\; & \approx x_{i}\left(k\right)-x_{i}\left(n\right)-B\left|\Delta x_{p,i}\left(n\right)\right|\left[\begin{array}{c} -\sin A\\ \cos A \end{array}\right]+X_{k}-X_{p}-Y\left[\begin{array}{c} \cos A-B\sin A\\ \sin A+B\cos A \end{array}\right]\;\;\\ & \approx x_{i}\left(k\right)-x_{i}\left(n\right)+X_{k}-X_{p}-Y\left[\;\begin{array}{c} \cos A\\ \sin A \end{array}\;\right]-B\left(|\Delta x_{p,i}\left(n\right)|+Y\right)\left[\begin{array}{c} -\sin A\\ \cos A \end{array}\right].\; \end{array}$$

Since all random variables in Equation (A4) are zero-mean and mutually independent, the expected value of difference vector between RSE ${\tilde{x}}_{i}\left(k\right)$ and LSE ${\hat{x}}_{p,i}\left(n\right)$ is given by

(A5) $$E\left[{\tilde{x}}_{i}\left(k\right)-{\hat{x}}_{p,i}\left(n\right)\right]\approx x_{i}\left(k\right)-x_{i}\left(n\right).$$

Then, the first two diagonal entries of convariance matrix in Equation (A1) can be represented by

(A6) $$\begin{array}{cc} \left[\begin{array}{c} \sigma_{1}^{2}\\ \sigma_{2}^{2} \end{array}\right]\; & =E\left[{\left({\tilde{x}}_{i}\left(k\right)-{\hat{x}}_{p,i}\left(n\right)\right)}^{2}\right]-{\left\{E\left[{\tilde{x}}_{i}\left(k\right)-{\hat{x}}_{p,i}\left(n\right)\right]\right\}}^{2}\;\;\\ & \approx \sigma_{X}^{2}1+\;\sigma_{Y}^{2}\left[\begin{array}{c} \cos^{2}A\\ \sin^{2}A \end{array}\right]+\left(\sigma_{\Theta }^{2}+\sigma_{\Phi }^{2}\right)\left(|\Delta x_{p,i}{\left(n\right)|}^{2}+\sigma_{Y}^{2}\right)\left[\begin{array}{c} \sin^{2}A\\ \cos^{2}A \end{array}\right], \end{array}$$

where $1={\left[1\;1\right]}^{⊤}$.

The difference of centrifugal speeds ${\tilde{\dot{x}}}_{p,i}^{C}\left(k\right)-{\hat{\dot{x}}}_{p,i}^{C}\left(n\right)$ is represented using Equations (13) and (16):

(A7) $$\begin{array}{cc} {\tilde{\dot{x}}}_{p,i}^{C}\left(k\right)-{\hat{\dot{x}}}_{p,i}^{C}\left(n\right)\; & =\frac{\left({\tilde{x}}_{i}\left(k\right)-{\tilde{x}}_{i}\left(p\right)\right)}{|{\tilde{x}}_{i}\left(k\right)-{\tilde{x}}_{i}\left(p\right)|}\cdot \left|{\tilde{\dot{x}}}_{i}\left(k\right)\right|\left[\begin{array}{c} \cos{\tilde{\theta }}_{i}\left(k\right)\\ \sin{\tilde{\theta }}_{i}\left(k\right) \end{array}\right]\;\\ & -|{\tilde{\dot{x}}}_{i}\left(p\right)|\cos\left(\phi_{p,i}\left(n\right)+\Phi \right)-\Delta {\dot{x}}_{p,i}^{C}\left(n\right)-Z. \end{array}$$

Since the first term of inner product in Equation (A7) is a point vector in a unit circle, its angle is represented by random variable $\Psi_{p,i}\left(k\right)$, i.e.,

$$\frac{\left({\tilde{x}}_{i}\left(k\right)-{\tilde{x}}_{i}\left(p\right)\right)}{|{\tilde{x}}_{i}\left(k\right)-{\tilde{x}}_{i}\left(p\right)|}=\left[\begin{array}{c} \cos{\tilde{\psi }}_{p,i}\left(k\right)\\ \sin{\tilde{\psi }}_{p,i}\left(k\right) \end{array}\right]=\left[\begin{array}{c} \cos(\phi_{p,i}\left(k\right)+\Psi_{p,i}\left(k\right))\\ \sin(\phi_{p,i}\left(k\right)+\Psi_{p,i}\left(k\right)) \end{array}\right].$$

Then, the inner product in Equation (A7) is

$$\frac{\left({\tilde{x}}_{i}\left(k\right)-{\tilde{x}}_{i}\left(p\right)\right)}{|{\tilde{x}}_{i}\left(k\right)-{\tilde{x}}_{i}\left(p\right)|}\cdot \left[\begin{array}{c} \cos{\tilde{\theta }}_{i}\left(k\right)\\ \sin{\tilde{\theta }}_{i}\left(k\right) \end{array}\right]=\left[\begin{array}{c} \cos{\tilde{\psi }}_{p,i}\left(k\right)\\ \sin{\tilde{\psi }}_{p,i}\left(k\right) \end{array}\right]\cdot \left[\begin{array}{c} \cos{\tilde{\theta }}_{i}\left(k\right)\\ \sin{\tilde{\theta }}_{i}\left(k\right) \end{array}\right]=\cos\left(C+D\right),$$

where $C=\theta_{i}\left(k\right)-\phi_{p,i}\left(k\right)$ and $D=\Theta_{k}-\Psi_{p,i}\left(k\right)$. For the simplicity of computation, $\Psi_{p,i}\left(k\right)$ is assumed to be zero-mean with variance of $\sigma_{\Psi_{p,i}\left(k\right)}^{2}$, which is estimated by the sample variance. Random variable $\Psi_{p,i}\left(k\right)$ is also assumed to be independent of all other random variables. Then, the difference of centrifugal speeds in Equation (A7) is represented by

(A8) $$\begin{array}{cc} {\tilde{\dot{x}}}_{p,i}^{C}-{\hat{\dot{x}}}_{p,i}^{C}\left(n\right)\; & \approx |{\tilde{\dot{x}}}_{i}\left(k\right)|\cos\left(C+D\right)-|{\tilde{\dot{x}}}_{i}\left(p\right)|\cos\left(\phi_{p,i}\left(n\right)+\Phi_{n}\right)-\Delta {\dot{x}}_{p,i}^{C}\left(n\right)-Z\;\;\\ & \approx |{\dot{x}}_{i}\left(k\right)|\left\{\cos C-D\sin C\right\}-\left|{\dot{x}}_{i}\left(p\right)\right|\left(\cos\phi_{p,i}\left(n\right)-\Phi_{n}\sin\phi_{p,i}\left(n\right)\right)\;\\ & +V\left\{\cos C-D\sin C-\cos\phi_{p,i}\left(n\right)+\Phi_{n}\sin\phi_{p,i}\left(n\right)\right\}-\Delta {\dot{x}}_{p,i}^{C}\left(n\right)-Z. \end{array}$$

Since all random variables are zero-mean and mutually independent, the expectation of ${\tilde{\dot{x}}}_{p,i}^{C}-{\hat{\dot{x}}}_{p,i}^{C}\left(n\right)$ in Equation (A8) is represented by

(A9) $$E\left[{\tilde{\dot{x}}}_{p,i}^{C}-{\hat{\dot{x}}}_{p,i}^{C}\left(n\right)\right]\approx |{\dot{x}}_{i}\left(k\right)|\cos C-|{\dot{x}}_{i}\left(p\right)|\cos\phi_{p,i}\left(n\right)-\Delta {\dot{x}}_{p,i}^{C}\left(n\right).$$

Using Equations (A8) and (A9), the third diagonal entry in Equation (A1) is approximated by

(A10) $$\begin{array}{cc} \sigma_{3}^{2}\; & =E\left[{\left({\tilde{\dot{x}}}_{p,i}^{C}-{\hat{\dot{x}}}_{p,i}^{C}\left(n\right)\right)}^{2}\right]-{\left\{E\left[{\tilde{\dot{x}}}_{p,i}^{C}-{\hat{\dot{x}}}_{p,i}^{C}\left(n\right)\right]\right\}}^{2}\;\\ & \approx \left(\sigma_{\Theta }^{2}+\sigma_{\Psi_{p,i}\left(k\right)}^{2}\right)|{\dot{x}}_{i}{\left(k\right)|}^{2}\sin^{2}C+\sigma_{\Phi }^{2}{\left|{\dot{x}}_{i}\left(p\right)\right|}^{2}\sin^{2}\phi_{p,i}\left(n\right)+\sigma_{V}^{2}{\left(\cos C-\cos\phi_{p,i}\left(n\right)\right)}^{2}\;\\ & +\sigma_{V}^{2}\left(\sigma_{\Theta }^{2}+\sigma_{\Psi_{p,i}\left(k\right)}^{2}\right)\sin^{2}C+\sigma_{V}^{2}\sigma_{\Phi }^{2}\sin^{2}\phi_{p,i}\left(n\right)+\sigma_{Z}^{2}. \end{array}$$

We next derive the nondiagonal entries $\sigma_{i,j}^{2}$ of covariance matrix in Equation (A1). Denoting by ${\hat{x}}_{p,i}\left(n\right)={\left[{\hat{x}}_{p,i}\left(n\right)\;\;\;{\hat{y}}_{p,i}\left(n\right)\right]}^{⊤}$ and ${\tilde{x}}_{i}\left(k\right)={\tilde{x}}_{i}\left(k\right)={\left[{\tilde{x}}_{i}\left(k\right)\;\;\;{\tilde{y}}_{i}\left(k\right)\right]}^{⊤}$ in Equation (A2), the covariance $\sigma_{1,2}^{2}$ between *x-axis and* y-axis position differences is approximated using Equations (A4) and (A5), as follows:

(A11) $$\begin{array}{cc} \sigma_{1,2}^{2}\; & =E\left[\left({\hat{x}}_{p,i}\left(n\right)-{\tilde{x}}_{i}\left(k\right)\right)\left({\hat{y}}_{p,i}\left(n\right)-{\tilde{y}}_{i}\left(k\right)\right)\right]-E\left[{\hat{x}}_{p,i}\left(n\right)-{\tilde{x}}_{i}\left(k\right)\right]E\left[{\hat{y}}_{p,i}\left(n\right)-{\tilde{y}}_{i}\left(k\right)\right]\;\\ & \approx \left[\sigma_{Y}^{2}-\left(\sigma_{\Theta }^{2}+\sigma_{\Phi }^{2}\right)\left(|\Delta x_{p,i}{\left(n\right)|}^{2}+\sigma_{Y}^{2}\right)\right]\cos A\sin A. \end{array}$$

Using Equations (A4), (A5), (A8), and (A9), the covariances $\sigma_{1,3}^{2}$ and $\sigma_{2,3}^{2}$ are approximated by

(A12) $$\begin{array}{cc} \sigma_{1,3}^{2}\; & =E\left[\left({\hat{x}}_{p,i}\left(n\right)-{\tilde{x}}_{i}\left(k\right)\right)\left({\tilde{\dot{x}}}_{p,i}^{C}-{\hat{\dot{x}}}_{p,i}^{C}\left(n\right)\right)\right]-E\left[{\hat{x}}_{p,i}\left(n\right)-{\tilde{x}}_{i}\left(k\right)\right]E\left[{\tilde{\dot{x}}}_{p,i}^{C}-{\hat{\dot{x}}}_{p,i}^{C}\left(n\right)\right]\;\\ & \approx \sigma_{\Phi }^{2}|\Delta x_{p,i}\left(n\right)||{\dot{x}}_{i}\left(p\right)|\sin A\sin\phi_{p,i}\left(n\right), \end{array}$$

and

(A13) $$\begin{array}{cc} \sigma_{2,3}^{2}\; & =E\left[\left({\hat{y}}_{p,i}\left(n\right)-{\tilde{y}}_{i}\left(k\right)\right)\left({\tilde{\dot{x}}}_{p,i}^{C}-{\hat{\dot{x}}}_{p,i}^{C}\left(n\right)\right)\right]-E\left[{\hat{y}}_{p,i}\left(n\right)-{\tilde{y}}_{i}\left(k\right)\right]E\left[{\tilde{\dot{x}}}_{p,i}^{C}-{\hat{\dot{x}}}_{p,i}^{C}\left(n\right)\right]\;\\ & \approx -\sigma_{\Phi }^{2}|\Delta x_{p,i}\left(n\right)||{\dot{x}}_{i}\left(p\right)|\cos A\sin\phi_{p,i}\left(n\right), \end{array}$$

respectively.

## Appendix B. Simulation of Exteroceptive Sensing in Vehcle Sensing Simulator (VSS)

In this Appendix, we describe the basic idea of simulating the exteroceptive sensing based on the radar model in Section 3.2. Without loss of generality, we focus on the construction of set $N_{p,i}$ for an arbitrary pivot vehicle *p* in Figure A1.

For each pivot vehicle, the VSS internally maintains the set of blocked angles $\Omega_{p,i}$ and the set of sensed vehicles $N_{p,i}$, where both sets are initialized to be empty set. Given the SUMO trace having the ground truth of every vehicle position $x_{i}\left(v\right)$, the VSS first constructs the list of surrounding vehicles whose distances from pivot vehicle *p* are no greater than LSR $D_{LS}$. Then, the surrounding vehicles are visited in the ascending order of the distance, for example 1, 3, 2, and 4 in Figure A1. For each surrounding vehicle *v*, the pivot vehicle calculates the interval of sensing angle $I_{\Phi }\left(v\right)=\;\left(\Delta \phi_{p,i}^{\min}\left(v\right),\Delta \phi_{p,i}^{\max}\left(v\right)\right)$ by inspecting the sensing angle of its four corners. To check whether vehicle *v* is obstructed by other vehicles or not, the VSS subtracts each interval $I_{\Phi }\left(\cdot \right)\in \Omega_{p,i}$ from $I_{\Phi }\left(v\right)$. If there exists an interval whose width is wider than angular resolution η in the subtracted result, vehicle *v* is inserted into the set of sensed vehicles $N_{p,i}$, as shown in Figure A1a,b,d. Otherwise, vehicle *v* is not inserted to $N_{p,i}$ as shown in Figure A1c. Next, set $\Omega_{p,i}$ is updated by the union of new interval $I_{\Phi }\left(v\right)$ and the overlapped interval(s) in set $\Omega_{p,i}$. This procedure is repeated until the VSS visits all surrounding vehicles, and returns the set of sensed vehicles, e.g., $N_{p,i}=\left\{1,3,4\right\}$ in Figure A1d.
